# Effect and cost-effectiveness of pneumococcal conjugate vaccination: a global modelling analysis

**DOI:** 10.1016/S2214-109X(18)30422-4

**Published:** 2018-12-13

**Authors:** Cynthia Chen, Francisco Cervero Liceras, Stefan Flasche, Sucitro Sidharta, Joanne Yoong, Neisha Sundaram, Mark Jit

**Affiliations:** a Saw Swee Hock School of Public Health, National University of Singapore, Singapore; b Modelling and Economics Unit, Public Health England, London, UK; c Center for Economic and Social Research, University of Southern California, Washington, DC, USA; d Department of Infectious Disease Epidemiology, London School of Hygiene & Tropical Medicine, London, UK; e Department of Global Health and Development, London School of Hygiene & Tropical Medicine, London, UK

## Abstract

**Background:**

Introduction of pneumococcal conjugate vaccines (PCVs) has substantially reduced disease burden due to *Streptococcus pneumoniae*, a leading cause of childhood morbidity and mortality globally. However, PCVs are among the most expensive vaccines, hindering their introduction in some settings and threatening sustainability in others. We aimed to assess the effect and cost-effectiveness of introduction of 13-valent PCV (PCV13) vaccination globally.

**Methods:**

We assessed the incremental cost-effectiveness ratio of PCV13 introduction by integrating two models: an ecological model (a parsimonious, mechanistic model validated with data from post-seven-valent PCV introduction in 13 high-income settings) to predict the effect of PCV on childhood invasive pneumococcal disease, and a decision-tree model to predict a range of clinical presentations and economic outcomes under vaccination and no-vaccination strategies. The models followed 30 birth cohorts up to age 5 years in 180 countries from 2015 to 2045. One-way scenario and probabilistic sensitivity analyses were done to explore model uncertainties.

**Findings:**

We estimate that global PCV13 use could prevent 0·399 million child deaths (95% credible interval 0·208 million to 0·711 million) and 54·6 million disease episodes (51·8 million to 58·1 million) annually. Global vaccine costs (in 2015 international dollars) of $15·5 billion could be partially offset by health-care savings of $3·19 billion (2·62 billion to 3·92 billion) and societal cost savings of $2·64 billion (2·13 billion to 3·28 billion). PCV13 use is probably cost-effective in all six UN regions. The 71 countries eligible for support from Gavi, the Vaccine Alliance, account for 83% of PCV13-preventable deaths but only 18% of global vaccination costs. The expected cost of PCV vaccination globally is around $16 billion per year.

**Interpretation:**

Our findings highlight the value of Gavi's support for PCV introduction in low-income countries and of efforts to improve the affordability of PCVs in countries not eligible for, or transitioning from, Gavi support.

**Funding:**

World Health Organization; Gavi, the Vaccine Alliance; and the Bill & Melinda Gates Foundation.

## Introduction

Pneumonia is the single largest global cause of mortality from infectious disease in children younger than 5 years.^1^ In 2015, pneumonia killed an estimated 920 000 children (14·9% of all deaths in children younger than 5 years).^2^ *Streptococcus pneumoniae* and *Haemophilus influenzae* type b are the leading causative organisms for pneumonia deaths, as well as a substantial proportion of deaths due to meningitis and sepsis.^3^, ^4^

Immunisation is the most effective means of prevention, and introduction of *H influenzae* type b vaccination in almost all countries has led to sharp reductions in *H influenzae*-attributable pneumonia over the past decade.^5^ Two pneumococcal conjugate vaccines (PCVs), ten-valent PCV (PCV10) and 13-valent PCV (PCV13), are also used in children. These PCVs have been widely adopted in high-income countries and have also been introduced in low-income countries with the support of Gavi, the Vaccine Alliance. However, more than 40 countries have yet to introduce PCVs into their national immunisation schedules.^6^ These countries are mostly located in Asia and Africa, where disease burden is highest, and include several countries with large populations, such as China and Nigeria. In addition, many low-income countries that have introduced PCVs are transitioning from eligibility for Gavi support and will therefore need to fund vaccination from national health-care budgets and, eventually, at higher prices. Enumerating the potential benefits, long-term budget implications, and cost-effectiveness of PCVs is crucial to sustain funding for these vaccines in countries that have introduced them, and to assess their introduction in other countries.

However, for many countries, the cost-effectiveness of PCV introduction remains to be evaluated rigorously and in an internationally comparable manner.^7^, ^8^ Existing studies either omit many of the effects of PCV introduction, such as herd protection and serotype replacement, or address only a subset of countries.^9^, ^10^

To address this evidence gap, we evaluated the cost-effectiveness of introducing PCV13 vaccination globally, accounting for regional pneumococcal epidemiology and using post-vaccination data from countries that have introduced PCVs.

**Research in context**

**Evidence before this study**

We searched PubMed without language or date restrictions for all records matching “(pneumococcal conjugate vaccine) and (cost-effectiveness) and (child*) and (global)” in any field. Our review identified 22 cost-effectiveness evaluations. We also examined articles identified in two reviews of pneumococcal conjugate vaccine (PCV) cost-effectiveness. We found three articles that estimated the impact of PCVs in low-income and middle-income countries, but no study assessing the cost-effectiveness of PCVs globally.

**Added value of this study**

Our analysis combines an ecological model, validated to post-vaccination data from 13 sites, with an economic model projecting health and economic outcomes of vaccination. It is the first study to integrate ecological and economic modelling to assess the impact and cost-effectiveness of PCVs in 180 countries. We show that vaccination is cost effective in almost every country, according to both highly cited gross-domestic-product-based thresholds and country-specific cost-effectiveness thresholds derived using supply-side considerations.

**Implications of all the available evidence**

PCV introduction is likely to be a cost-effective way to save children's lives and avert disability in all UN regions and most countries. The benefits are particularly large in Gavi-eligible countries and in Africa and Asia. However, vaccine introduction requires an increased financial investment, which is partially offset by health-care cost savings from reduced disease. Thus, these findings underscore the importance of Gavi support and of mechanisms to support vaccine introduction at affordable prices for disadvantaged populations not eligible for Gavi support.

## Methods

### Model overview and framework

The cost-effectiveness of PCV introduction was assessed by linking two models. The first, an ecological model, predicted the effect of PCVs on childhood invasive pneumococcal disease (IPD); the term ecological is used here in the epidemiological rather than biological sense, wherein the unit of analysis is the population rather than the individual. The second model, a decision-tree model, used outputs of the ecological model to predict a range of clinical presentations and economic outcomes. The combined model followed 30 birth cohorts of children younger than 5 years from 2015 to 2045 in 180 countries.

### Ecological model

The ecological model was a parsimonious mechanistic model that has been previously validated to post-PCV7 data from 13 high-income settings.^11^ The model simplified the long-term impact predictions, including serotype replacement and herd protection, from more elaborate susceptible-infectious-susceptible-type dynamic transmission models into a single predictive equation by making a number of assumptions: that vaccine serotypes will eventually be eliminated as a result of PCV use; that eliminated serotypes will be fully replaced in carriage by non-vaccine serotypes; and that the propensity of non-vaccine serotypes to cause invasive disease if carried remains the same in the post-PCV era. On this basis, the predicted incidence risk ratio is

$$\text{Incidence risk ratio}=\frac{\text{c}+1}{\text{d}+1}$$

where c is the odds of carriage due to vaccine serotypes and d is the odds of IPD. Given the assumption of vaccine serotype elimination, model predictions should be treated as estimates of the maximum reduction in IPD that can be achieved through vaccination, rather than necessarily predictions of vaccine impact. In particular, in settings with low vaccine coverage or intense transmission, vaccination might not be able to completely eliminate vaccine serotypes. Therefore, we present a sensitivity analysis based on the assumption that reduction in carriage due to vaccine serotypes is only 65% (as observed in Kilifi, Kenya^12^), and PCV impact is thus only 65% of our model's base-case prediction.

We separately parameterised the model for six UN regions (Africa, Asia, Europe, Latin American and the Caribbean, North America, and Oceania) and assumed PCV impact to be the same in each region. We used regional PCV13 serotype coverage among paediatric IPD isolates before routine use of PCVs, as reported in a 2010 global meta-analysis.^13^ Similarly, we used the PCV13 serotype coverage among paediatric carriage as reported in another, unpublished, global meta-analysis (le Polain de Waroux O and Flasche S, London School of Hygiene & Tropical Medicine, personal communication). We used 1000 posterior samples for PCV13 serotype coverage from the latter study. For IPD, because no posterior distributions were reported, we fitted a binomial distribution to the reported regional PCV13 serotype coverage estimates to match reported means and 95% confidence intervals, and took 1000 bootstrap samples from those. Using the formula above, estimates of PCV serotype coverage in carriage and IPD were combined to obtain 1000 posterior samples of the regional effect of PCV13 on paediatric IPD, which were used in the economic model.

To extend the outcomes of the model, we assumed that the proportion of vaccine-preventable non-invasive pneumococcal disease (nIPD) was the same as that for IPD (details on model structure are shown in the appendix).^11^, ^14^, ^15^ Furthermore, we assumed that use of PCVs would reduce all-cause acute otitis media incidence by 19%, as estimated in the COMPAS trial.^16^ It was also assumed that the proportionate reduction in disease over time after vaccination follows the same time course as that observed in a multi-country review of post-vaccination data^14^ (ie, that 79% of the impact is achieved in the first year following introduction of PCV, and the full impact is established from year 2 onwards).

### Economic model

**Figure 1:**
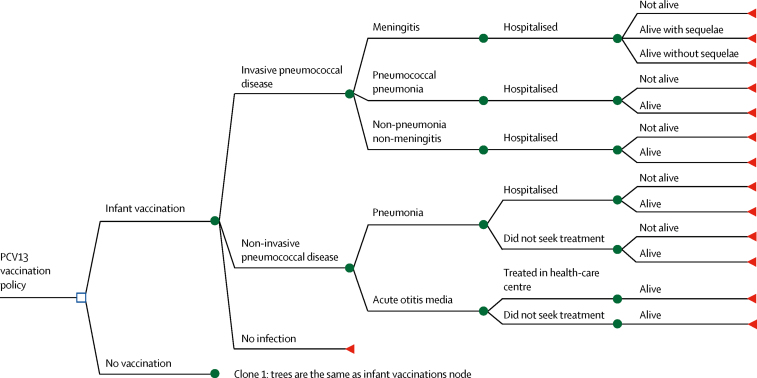
Decision tree for outcomes over a single year of age, depicting vaccination with PCV13 versus no vaccination An age-stratified decision-tree economic model was developed to represent disease outcomes and associated health states for vaccinated and unvaccinated populations in the model. The same structure is repeated for every year of age between 0 and 5 years. PCV13=13-valent pneumococcal conjugate vaccine.

The decision-tree model (figure 1) represented disease outcomes and associated health states under both strategies, and assessed risk of various clinical presentations of IPD and nIPD: meningitis, pneumonia, non-pneumonia non-meningitis, and acute otitis media.

In the vaccination strategy, we used national immunisation schedules reported by WHO for 128 countries, with either three (2 + 1, 3 + 0) or four (3 + 1) doses, and assumed regional schedules for countries without information from WHO. Vaccine coverage was assumed equal to national three-dose diphtheria-tetanus-pertussis vaccine (DTP3) coverage.

Disease burden for the various presentations was informed by meta-analyses and global estimates^3^, ^17^, ^18^, ^19^ (details of model parameterisation are shown in the appendix). The regional values resulting from incidence and case-fatality risk distributions were applied to individual countries; for the proportion of invasive pneumonia and the risk of major sequelae, single values from global meta-analyses were used and applied to all regions.

Disease could lead to hospital admission, treatment at a health centre, or not seeking care, depending on condition. The resulting health outcomes were complete recovery, recovering with sequelae for meningitis, or death. Each episode was associated with a disability weight and cost. The disability due to each disease episode, as well as meningitis sequelae, was estimated in terms of disability-adjusted life-years (DALYs) from the WHO Global Burden of Disease (GBD) 2000 study.^20^

We included vaccination and health-care costs (health-system costs), as well as out-of-pocket expenditures and productivity losses. Vaccination costs included vaccine purchase, incremental costs of vaccine introduction activities and cold chain needs, and vaccine administration. For high-income countries, we used a price per dose of US$120·39, based on the price paid by the Vaccines for Children programme in the USA.^21^ For other non-Gavi-eligible countries, the price per dose was assumed to be $15·68, based on the procurement prices of the Pan-American Health Organization Revolving Fund.^22^ For low-income and other Gavi-eligible countries, we used a price of $3·05 (plus 20% tail price) per dose in accordance with the Gavi Advance Market Commitment.^23^ A freight cost of 6% of the vaccine price, a 5% wastage rate, and a buffer stock of 25% of the first-year vaccine needs were included. Vaccine administration costs included a nurse's time to administer the vaccine and related injection supplies.

Health-care costs included treatment in hospital for all disease presentations except acute otitis media, for which outpatient costs were used. We accounted for care-seeking behaviour for pneumonia and acute otitis media. Any health-care costs for non-invasive pneumonia that did not result in treatment in hospital were not included in our model. Costs in low-income and middle-income countries for meningitis were extracted from the report by Portnoy and colleagues.^24^ Costs for pneumonia were modelled from data in the WHO-CHOICE database^25^ and a 2016 systematic review.^26^ Other costs were estimated from predictions of best-fit models, which we parameterised using data for individual countries extracted from 27 studies of the cost-effectiveness of PCVs. To reflect the economic burden borne by the households and society, we estimated out-of-pocket expenditures that families with sick children would have to incur and the productivity losses due to loss of work days for the main carer, assumed to be the mothers, using values from the WHO global health expenditure database and female labour force participation rates for each country (appendix).

All unit prices were converted to 2015 international dollars (I$). Models were coded in R version 3.3.1 and Microsoft Excel version 15.37.

### Economic evaluation

We evaluated the costs and benefits of introducing the vaccine in countries currently without PCV programmes and in countries that are eligible for Gavi's Advanced Market Commitment. The incremental cost-effectiveness ratio (ICER) of PCV introduction was defined as the discounted incremental cost of PCV introduction over 30 years divided by the discounted incremental DALYs averted by vaccination over 30 years. To calculate ICER, future costs and outcomes were discounted at 3% per annum.

We did a budget impact analysis to identify the financial consequences of vaccination. We estimated the financing flows showing the fiscal impact of vaccines globally. The projections of the budget impact analysis were intended to provide guidance while assessing the affordability of PCV introduction.

To assess whether PCV introduction was cost-effective in a particular setting, we compared the ICER to two thresholds: gross domestic product (GDP) at purchasing power parity per capita (the threshold suggested by the Commission for Macroeconomics and Health for an intervention to be “very cost-effective”^27^); and country-specific thresholds, published by Woods and colleagues, based on extrapolating the opportunity costs of health-care spending in the UK.^28^

### Sensitivity analysis

One-way scenario and probabilistic sensitivity analyses were done to test the robustness of our model results to changes in key parameters over plausible ranges.

For one-way analyses, we assessed the effect of varying disease incidence and case fatality rates of all disease presentations between the lower and upper 95% uncertainty bounds reported in the three systematic reviews.^3^, ^17^, ^29^ The effects of varying cost inputs on the ICER were assessed by varying vaccine price and health-care costs by ±20%. Discounts were varied between 0% and 6%. The ratio of IPD to nIPD was also varied between 0·022 and 0·509 as per the Gambia study by Cutts and colleagues.^29^

We also did scenario sensitivity analyses using disability values from the GBD 2015 study;^30^ WHO-CHOICE costs and estimated lengths of stay for health-care costs; excluding countries with low vaccination coverage (DTP3 <70%) from PCV introduction; and assuming that low-income and middle-income countries achieve only a 65% reduction of vaccine serotype carriage (instead of full elimination), as shown in a post-introduction study in Kenya.^12^

A second-order probabilistic sensitivity analysis of the statistical uncertainty of parameters was done with Monte Carlo simulation, and the model was simulated 1000 times with bootstrap samples, with replacement from probability distributions of parameters, to explore the effect of statistical uncertainty on study results. Disease incidence and case fatality rates were sampled from distributions. Estimates of costs, magnitude of ecological effects, and ICERs were obtained for each sample, allowing us to generate 95% credible intervals.

### Role of the funding source

The funder of the study had no role in study design, data collection, data analysis, data interpretation, or writing of the report. The corresponding author had full access to all the data in the study and had final responsibility for the decision to submit for publication.

## Results

In our global analysis of 180 countries, we project more than 1·18 million deaths (95% credible interval 0·780 million to 1·76 million) and 457 million disease episodes (449 million to 465 million) annually before vaccination in children younger than 5 years. Vaccination could prevent 34% of global deaths (0·399 million [0·208 million to 0·711 million]) and 12% of disease episodes (54·6 million [51·8 million to 58·6 million]). The largest benefits would be seen in Africa (0·275 million deaths averted [0·114 million to 0·567 million] and 8·68 million DALYs averted [3·99 million to 16·8 million]), followed by Asia (0·0923 million deaths averted [0·0340 million to 0·176 million] and 3·88 million DALYs averted [1·75 million to 6·78 million]) a year. Vaccination in all other regions would collectively avert 0·0238 million deaths (0·0152 million to 0·0358 million) and 0·979 million DALYs (0·699 million to 1·38 million) annually (table, appendix).

**Table:** Median yearly global and regional estimated incremental outcomes of PCV13 vaccination compared with no vaccination

	Global*	**Africa**	**Asia**	**Oceania**	**Europe**	**Latin America and the Caribbean**	**North America**
Number of children fully vaccinated with at least three doses (millions)	114	34·6	58·1	0·559	6·73	8·77	4·42
Vaccination programme costs (undiscounted; I$, billions)	15·5	1·80	6·06	0·144	4·03	1·11	2·39
Health-care costs (undiscounted; I$, billions)	−3·19 (−3·92 to −2·62)	−0·364 (−0·507 to −0·239)	−1·35 (−1·91 to −0·857)	−0·0637 (−0·101 to −0·0235)	−0·380 (−0·441 to −0·329)	−0·222 (−0·266 to −0·188)	−0·810 (−0·960 to −0·681)
Societal costs (undiscounted; I$, billions)	−2·64 (−3·28 to −2·13)	−0·463 (−0·619 to −0·325)	−1·46 (−2·02 to −0·975)	−0·0297 (−0·0419 to −0·0168)	−0·237 (−0·261 to −0·217)	−0·182 (−0·215 to −0·158)	−0·262 (−0·296 to −0·233)
Invasive pneumococcal disease cases (millions)	−1·65 (−2·48 to −0·986)	−0·757 (−1·31 to −0·303)	−0·725 (−1·26 to −0·270)	−0·00781 (−0·0134 to −0·00234)	−0·0280 (−0·0371 to −0·0203)	−0·0696 (−0·0923 to −0·0518)	−0·0487 (−0·0624 to −0·0365)
Non-invasive pneumococcal disease cases (millions)	−4·14 (−6·24 to −2·48)	−1·82 (−3·10 to −0·740)	−1·94 (−3·39 to −0·721)	−0·0206 (−0·0343 to −0·00629)	−0·0622 (−0·0816 to −0·0458)	−0·151 (−0·196 to −0·116)	−0·105 (−0·133 to −0·0813)
Acute otitis media cases (millions)	−48·8 (−49·4 to −48·3)	−24·5 (−24·7 to −24·3)	−19·2 (−19·5 to −18·9)	−0·266 (−0·269 to −0·263)	−1·85 (−1·88 to −1·83)	−1·92 (−1·96 to −1·88)	−1·12 (−1·13 to −1·11)
Deaths (millions)	−0·399 (−0·711 to −0·208)	−0·275 (−0·567 to −0·114)	−0·0923 (−0·176 to −0·0340)	−0·000611 (−0·00123 to −0·000175)	−0·00486 (−0·00809 to −0·00276)	−0·0108 (−0·0157 to −0·00713)	−0·00754 (−0·0108 to −0·00510)
DALYs averted by PCV13 (undiscounted; millions)	13·8 (8·08–23·0)	8·68 (3·99–16·8)	3·88 (1·75–6·78)	0·0328 (0·0141–0·0556)	0·216 (0·149–0·320)	0·429 (0·313–0·588)	0·301 (0·222–0·415)
DALYs averted by PCV13 (discounted; millions)	9·13 (5·33–15·0)	5·65 (2·59–10·9)	2·62 (1·18–4·58)	0·0217 (0·00929–0·0369)	0·146 (0·100–0·216)	0·291 (0·212–0·399)	0·200 (0·148–0·276)
Total incremental costs from PCV13 (undiscounted; I$, billions)	9·71 (8·33–10·8)	0·977 (0·677–1·24)	3·25 (2·15–4·23)	0·0503 (0·000356–0·103)	3·41 (3·32–3·48)	0·707 (0·632–0·767)	1·32 (1·13–1·48)
Total incremental costs from PCV13 (discounted; I$, billions)	6·67 (5·74–7·40)	0·663 (0·466–0·836)	2·26 (1·51–2·92)	0·0345 (0·00121–0·0698)	2·34 (2·29–2·39)	0·488 (0·437–0·529)	0·886 (0·763–0·992)
Total health-system costs† (undiscounted; I$, billions)	12·3 (11·6–12·9)	1·44 (1·30–1·56)	4·71 (4·15–5·20)	0·0800 (0·0423–0·120)	3·65 (3·59–3·70)	0·890 (0·846–0·925)	1·58 (1·43–1·71)
Total health-system costs† (discounted; I$, billions)	8·42 (7·93–8·81)	0·962 (0·868–1·04)	3·24 (2·86–3·57)	0·0541 (0·0289–0·0809)	2·50 (2·46–2·54)	0·611 (0·581–0·635)	1·06 (0·959–1·14)
Incremental cost-effectiveness ratio over 30 years‡ (I$ per DALYs averted)	724 (400–1360)	118 (45·7–320)	853 (340–2450)	1590 (36·7–7560)	16 000 (10800–23700)	1680 (1120–2420)	4420 (2880–6440)

Values are point estimate (95% credible interval) and are shown to 3 significant figures. Negative values indicate costs saved, or cases or deaths averted. PCV13=13-valent pneumococcal conjugate vaccine. I$=international dollars. DALYs=disability-adjusted life-years.

* Because of rounding differences, global values are only approximately equal to the sum of values for individual regions.

† Health-system costs include vaccination programme costs and health-care costs; societal costs (out-of-pocket expenses and productivity costs) are excluded.

‡ Because of rounding differences, values are only approximately equal to total incremental costs from PCV13 (discounted) divided by DALYs averted by PCV13 (discounted).

Pneumococcal disease in the absence of vaccination was associated with estimated global health-system costs of I$13·7 billion (12·9 billion to 14·7 billion) and societal costs of $14·3 billion (13·7 billion to 15·1 billion). We estimated a total global vaccination cost of $15·5 billion, or roughly $137 per fully vaccinated child. The cost per fully vaccinated child would be $541 in North America, $599 in Europe, $104 in Asia, and $52 in Africa. The higher total cost per vaccinated child in Europe (despite a four-dose schedule in North America) was due to a higher price per dose in Europe when costs were converted to GDP at purchasing power parity. Global investment would be concentrated in Asia ($6·06 billion; 39%), followed by Europe ($4·03 billion; 26%) and North America ($2·39 billion; 15%). Comparatively, Africa would require a smaller investment of $1·8 billion (12%) because of the wider availability of Gavi prices.

From the health-systems perspective, investment in vaccination would be partially compensated by savings of $3·19 billion (2·62 billion to 3·92 billion) from averted hospital inpatient care and health-centre visits globally. Societal cost savings would further offset the cost of vaccine introduction by an additional $2·64 billion (2·13 billion to 3·28 billion). These savings comprise productivity gains from reduced caregiving ($0·701 billion [0·617 billion to 0·807 billion]) and reduced out-of-pocket expenditures ($1·94 billion [1·49 billion to 2·49 billion]). Asia ($1·46 billion [0·975 billion to 2·02 billion]) and Africa ($0·463 billion [0·325 billion to 0·619 billion]) would together account for 72·9% of societal savings.

**Figure 2:**
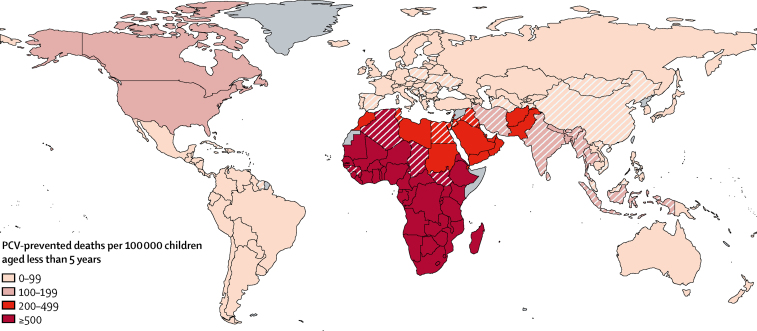
Estimated deaths prevented by PCV vaccination per 100 000 children younger than 5 years in 180 countries The map represents the number of deaths prevented by routine childhood vaccination with PCV at 2015 coverage levels compared with the no vaccination scenario. Countries that have implemented PCV programmes are shaded with solid colours. Countries without existing PCV programmes are shown with diagonal lines. Countries in grey (n=17) were excluded because of missing data. PCV=pneumococcal conjugate vaccine.

The 52 countries that do not have existing PCV vaccination programmes include countries that have high rates of PCV-preventable deaths such as Algeria, Egypt, Chad, and South Sudan (figure 2). We estimate that introduction of PCV in these 52 countries would prevent 90 000 deaths (44 800–154 000) and 18·4 million disease episodes (16·9 million to 20·2 million). Although vaccination in these countries would be cost-effective, at a median ICER of I$598 (244–1420) per DALY averted, PCV introduction would require a considerable investment of $4·42 billion. This amount is almost a third of the global costs.

**Figure 3:**
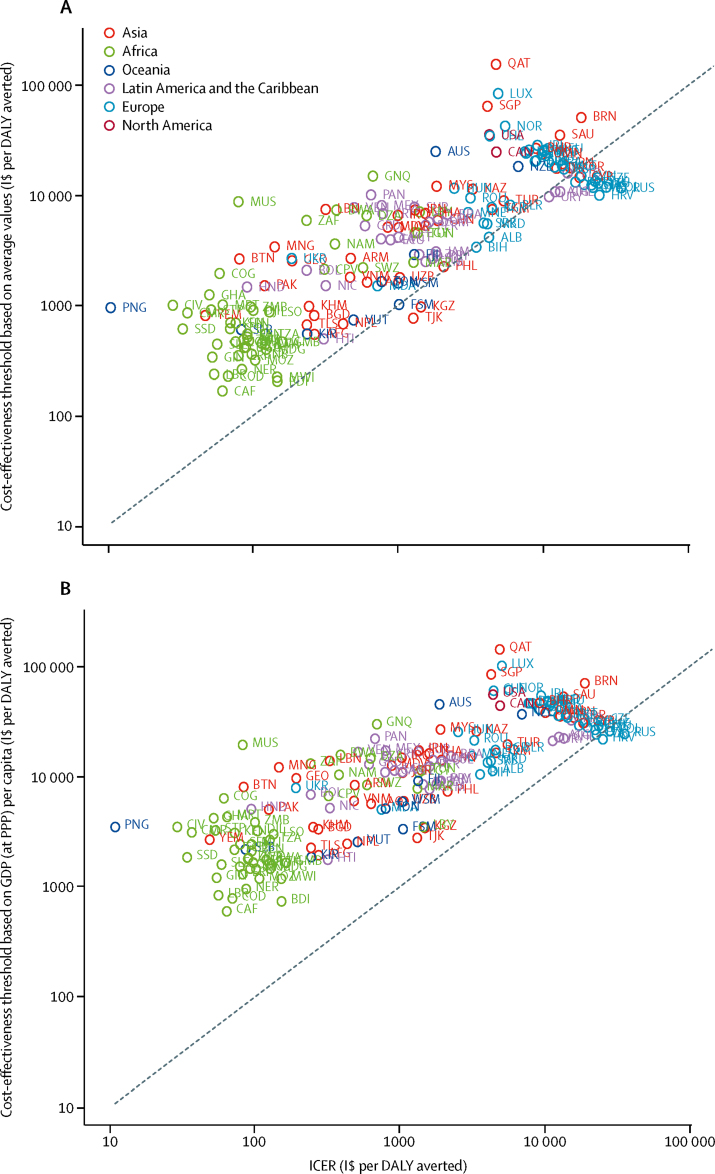
Cost-effectiveness of routine PCV13 childhood vaccination The graphs show ICERs of PCV13 vaccination versus no vaccination, by country, compared with cost-effectiveness thresholds based on average values reported by Woods and colleagues^28^ (A) and thresholds based on GDP (at PPP) per capita (B). The x-axis represents the cost-effectiveness estimate obtained from our model. Countries above the line (y=x) are cost-effective. Graphs are presented using a log-log scale. Credible intervals were omitted for clarity. DALY=disability-adjusted life-year. GDP=gross domestic product. ICER=incremental cost-effectiveness ratio. I$=international dollars. PCV13=13-valent pneumococcal conjugate vaccine. PPP=purchasing power parity.

**Figure 4:**
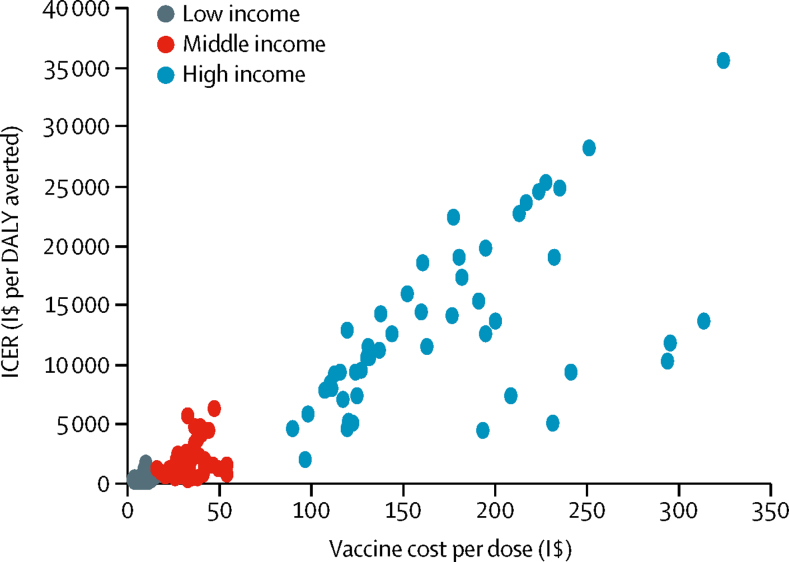
Comparison of ICER and vaccine purchase cost per dose by income regions Vaccine purchase cost per dose was converted to I$ using the World Bank and International Monetary Fund price level ratios of gross domestic product (at purchasing power parity) per capita to market exchange rate in 2015. Each point on the chart represents one country. Countries with higher vaccine cost had lower cost-effectiveness of vaccination (higher ICER). DALY=disability-adjusted life-year. ICER=incremental cost-effectiveness ratio. I$=international dollars.

Globally, PCV vaccination would cost $724 (400 to 1360; discounted) per DALY averted compared with no vaccination. Median ICER per DALY averted was estimated at $118 (45·7 to 320) in Africa, $853 (340 to 2450) in Asia, and $16 000 (10 800 to 23 700) in Europe. PCV introduction would be cost-saving in 11 countries (6%) and cost-effective in 154 countries (86%) using the thresholds according to Woods and colleagues,^28^ and cost-effective in 166 countries (92%) using GDP (at purchasing power parity) per capita thresholds (figure 3). Figure 4 shows how ICER changed with vaccine purchase cost by low-income, middle-income, and high-income regions.

Most of the effect of PCVs is from direct vaccine protection, although indirect effects also make an important contribution in Asia and Oceania (appendix). In North America, however, indirect effects are negative (ie, the total population effect is smaller than what would be expected based on summing the direct protection in each individual without herd protection or serotype replacement) because the predicted impact of serotype replacement is greater than that of herd effects.

The health and economic benefits of PCVs are concentrated among the 71 Gavi-eligible countries, because this group accounts for 83% of all lives saved (0·330 million [0·161 million to 0·616 million]) and only 18% ($2·80 billion) of the global investment needed. However, expanding PCV introduction among the 16 eligible countries that have not introduced it as of March, 2017, will require more intensive efforts than in the past: these countries (including India and Indonesia) account for 60 000 deaths (28 000–108 000), but will require almost half ($1·38 billion) the total investment required for all Gavi-eligible countries.

**Figure 5:**
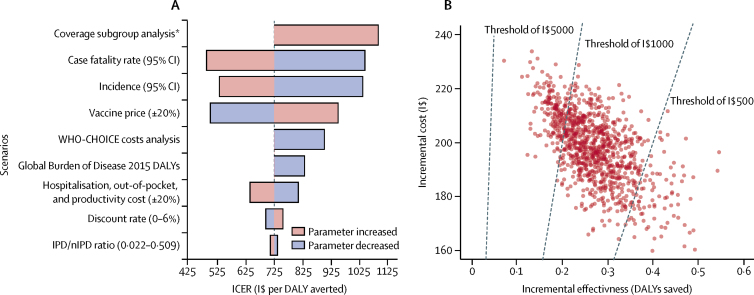
Results of one-way parameter scenario and probabilistic sensitivity analyses (A) One-way sensitivity analysis was done to test the robustness of the economic model by varying key parameters over plausible ranges (shown in parentheses) to assess their global effect on ICER and number of deaths. Bars represent the median ICER generated from 1000 bootstraps. Longer bars represent greater sensitivity of the global results to variations in that key parameter. (B) In the probabilistic sensitivity analysis diagram, each point represents the result of the incremental cost (y-axis), and effectiveness (x-axis) of one bootstrap sample on the global scale. A total of 1000 bootstraps were generated. 100% of the simulations resulted in a positive ICER (quadrant 1). Dotted lines indicate willingness-to-pay thresholds of I$500, $1000, and $5000 per DALY saved. Points to the right of each dotted line are cost-effective at that willingness-to-pay threshold. CI=credible interval. DALY=disability-adjusted life-year. ICER=incremental cost-effectiveness ratio. IPD=invasive pneumococcal disease. nIPD=non-invasive pneumococcal disease. I$=international dollars. *In the coverage subgroup analysis, 63 countries without national immunisation programmes or with three-dose diphtheria-tetanus-pertussis coverage of less than 70% were excluded.

Vaccination remained cost-effective under all one-way changes to key parameters in all regions. The results were most sensitive to variation in disease incidence and mortality parameters (figure 5A). The median pooled cost-effectiveness ratio varied between $487 (290–887) and $1090 (614–1950) per DALY averted. When the vaccine price was varied by 20%, the ICER changed by 31% in both directions. When WHO-CHOICE costs for health care were used, the ICER increased by 24%. When disability weights from GBD 2015 were used, the ICER increased by 15%, as these were lower per disease episode than the GBD 2000 figures. The results were least sensitive to discount rates and ratios of IPD to nIPD, with changes of less than 6%.

As a robustness check, we omitted countries without existing vaccine programmes or with DTP3 coverage less than 70%, assuming that complete elimination of vaccine-type *S pneumoniae* could not be achieved in these countries. PCV remained cost-effective, although the median ICER increased from $724 to $1090 per DALY averted. Similarly, in the scenario with only partial (65%) reduction in serotype carriage, the mean ICER increased only from $388 (full elimination) to $432 per DALY averted (with residual carriage) in low-income and middle-income countries; and globally, the mean ICER increased only from $724 (full elimination) to $831 per DALY adverted (with residual carriage).

In our sensitivity analysis (figure 5b), 100% of the simulations resulted in a positive ICER (ie, quadrant 1), indicating that, on the global scale, PCV vaccination is associated with greater life expectancy, although it comes at a higher cost. PCV vaccination was cost-effective in 84·7% of simulations when using a willingness-to-pay threshold of $1000 per DALY gained, and in 100% of simulations using a threshold of $5000 per DALY gained. However, using a more stringent willingness-to-pay threshold of $500 per DALY gained, PCV vaccination was cost-effective in 10·1% of simulations.

**Figure 6:**
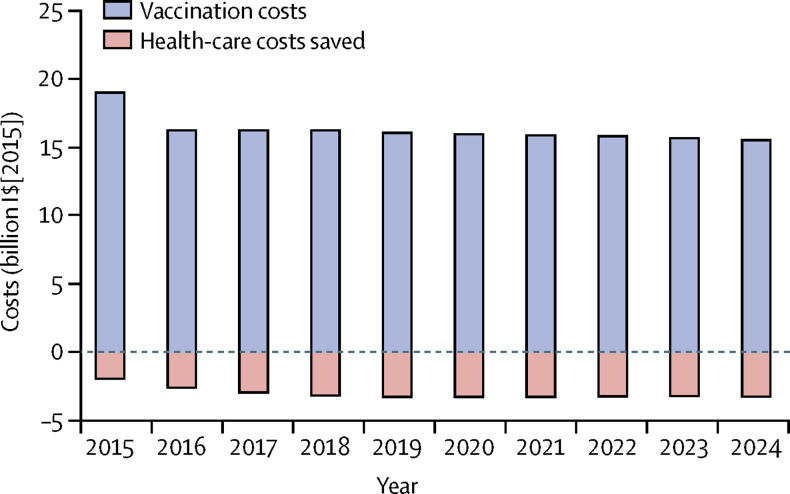
Global budget impact analysis of PCV vaccination over 10 years The budget impact analysis shows the effect of PCV vaccination on health-care costs (in 2015 I$). Negative values represent net savings in the health-care costs. Birth cohort size was assumed to vary based on UN population projection, and vaccine purchase costs were assumed to vary by countries' income classification. In the first birth cohort, we included a buffer stock of 25% and assumed it to remain constant over time. I$=international dollars. PCV=pneumococcal conjugate vaccine.

Figure 6 shows the effect of PCV vaccination on the health-care costs (in 2015 I$) from the provider's perspective. Birth cohort size was assumed to vary based on UN population projection, and vaccine purchase costs were assumed to vary by countries' income classification (appendix). In the first birth cohort, we included a buffer stock of 25%, assumed to remain constant with time. As such, the expected cost of PCV vaccination globally is $19 billion in the first year, and around $16 billion every year thereafter. The global vaccination programme is likely to reduce direct cost of health care by $1·95 billion (1·46 billion to 2·56 billion) in the first year and about $2·69 billion (2·08 billion to 3·46 billion) annually thereafter (appendix).

## Discussion

We predict that the introduction of PCV13 at DTP3 coverage levels in 180 countries could save around 0·4 million lives and avert 13·8 million DALYs annually among children younger than 5 years. These values include more than 90 000 additional lives saved in countries that had yet to introduce PCV as of Dec 1, 2016. Globally, PCVs could reduce the burden of pneumococcal disease (measured in terms of deaths, DALYs, and economic costs) most in the poorest regions. In particular, 92% of lives saved and 91% of DALYs averted in our simulations were in Africa and Asia.

The ICER for PCV introduction is less than GDP per capita in almost all regions and countries. The GDP per capita threshold has been traditionally used as an indication of cost-effectiveness,^31^ but has been criticised.^32^, ^33^ Using a more stringent threshold estimated by Woods and colleagues^28^ on the basis of the opportunity cost of health expenditure, PCV is cost-effective in 143 of 180 countries. PCV introduction throughout Africa requires only 12% of global PCV investments but accounts for 69% of the lives saved and 63% of the DALYs averted globally. PCV introduction at Advance Market Commitment prices offered to Gavi has the potential to support elimination of more than 80% of the global burden of vaccine-preventable pneumococcal disease, with a median ICER of $51·45 per DALY averted.

Our study is the first multi-country model to capture the ecological effects of PCV introduction (herd protection and serotype replacement) based on underlying serotype-specific rates of pneumococcal carriage. Previous economic evaluations have often ignored these effects or represented them using simplified assumptions, such as representing the indirect effects of PCV10 or PCV13 using the experience of PCV7 introduction in the USA.^7^ Therefore, our model shows the capabilities of an integrated ecological–economic model to evaluate the relative effect and cost-effectiveness of PCV on a global scale. To our knowledge, the only cost-effectiveness evaluations of PCV that consider more than a few countries were published in 2011 or earlier, and were restricted to Gavi-eligible countries alone.^9^, ^10^

Our analysis made several simplifying assumptions because of data and knowledge limitations. The ecological model assumed that vaccine coverage and effectiveness will be high enough to achieve elimination of vaccine serotypes in all schedules and coverage levels. High-income countries with serotype surveillance, such as the USA, the UK, and Australia, have reported near-elimination of IPD and pneumococcal carriage due to vaccine serotypes in most population groups following PCV introduction.^14^ Although some residual vaccine-type carriage following vaccination has been observed in African settings despite high vaccine coverage, we assumed that with such low residual vaccine-type carriage rates, our model would still perform better than other methods for predicting vaccine effect.

Because public sector vaccine tender prices are generally not documented, unless part of pooled procurement schemes, we extrapolated vaccine prices from a few settings with publicly available prices (Pan-American Health Organization Revolving Fund, Gavi, and the Vaccines for Children programme in the USA) to other countries in the same income group. This approach probably overestimates prices for high-income countries and underestimates prices in middle-income countries with no access to pooled procurement mechanisms. It is notable that most countries yet to introduce PCV into their routine schedules are middle-income countries outside Latin America (ie, those with no access to pooled procurement), possibly indicating that high vaccine prices are a barrier to wider introduction. Our analysis provides an indication of prices that countries could seek to negotiate for in national tenders, although country purchasers are usually encouraged to use their own nationally derived thresholds for such computations.^32^

Treatment costs were obtained from global meta-analyses or from the WHO-CHOICE database because of unavailability of cost-of-illness studies in most countries. Similarly, lifetime costs of meningitis sequelae, which can be substantial (as evidenced by a meta-analysis^24^ focused on low-income and middle-income countries), were not considered in this analysis because of the unavailability of cost-of-illness studies. Therefore, our analysis is conservative. However, our deterministic and probabilistic sensitivity analyses confirmed that PCV13 was cost-effective globally across changes in disease burden and treatment cost.

Finally, our study focused only on populations younger than 5 years. Infant vaccination protects older children and adults through herd effects, although post-introduction data in high-income countries suggest that reductions in vaccine-type disease in these groups are mitigated by increases in non-vaccine-type disease to a greater extent than in young children.^34^, ^35^ Our ability to model these dynamics is limited by the scarcity of data on serotype distribution in adult disease; however, the population effect of vaccination is likely to be higher if adults are included. For this reason, the aggregate cost-effectiveness of PCVs is an underestimate. Particularly in settings with a high disease burden among older individuals, as in most high-income countries, the indirect effect of vaccination on adult pneumococcal disease is likely to be significant.^36^

In conclusion, our results show large benefits of PCV use worldwide in terms of lives saved and disability averted, and in terms of cost-effectiveness, particularly in Africa and Asia. These results provide information for decision making in countries that have yet to introduce PCVs and countries that still have low coverage of existing PCV programmes. We also estimate a large financial cost associated with vaccine introduction that is only partially mitigated by treatment cost savings, and we acknowledge the risk for middle-income countries with no access to pooled procurement facing a financial barrier because of high vaccine prices. These findings call for renewed efforts on vaccine introduction support and the continuous pursuit of affordable prices for disadvantaged populations.
