# Supplementary material for: Effect and cost-effectiveness of pneumococcal conjugate vaccination: a global modelling analysis
Source: Lancet Glob Health. 2018 Dec 13;7(1):e58–67. doi: 10.1016/S2214-109X(18)30422-4 (PMC6293964; doi:10.1016/S2214-109X(18)30422-4)
Supplement: Supplementary appendix [file mmc1.pdf]

THE LANCET
Global Health

Supplementary appendix

This appendix formed part of the original submission and has been peer reviewed. We post it as supplied by the authors.

Supplement to: Chen C, Liceras FC, Flasche S, et al. Effect and cost-effectiveness of pneumococcal conjugate vaccination: a global modelling analysis. *Lancet Glob Health* 2019; **7:** e58–67.

METHODOLOGY AND RESULT APPENDIX

This appendix supplements the methodology and results of the main article. In particular, this appendix will present in more detail the study design and model structure, the parameters used for the model, and the various results of the model.

CONTENTS

METHODOLOGY AND RESULT APPENDIX 1

1. STUDY DESIGN AND MODEL STRUCTURE 2

   1.1. Study design 2

   1.2. Ecological vaccine impact 2

   1.3. Economic model 4

2. PARAMETERS 5

   2.1. Demographics 10

   2.2. Meningitis, Pneumonia, NPNM and Acute Otitis Media - Incidence and Case-Fatality Risks: 11

   2.3. Utilities 11

   2.4. Vaccination schedules and coverage 15

   2.5. Vaccination program cost 15

   2.6. Healthcare utilization 16

   2.7. Healthcare costs 17

   2.8. Societal costs 19

   2.9. Incremental Cost-Effectiveness Ratios – ICERs 19

   2.10. Sensitivity analysis 19

   2.11. Budget Impact Analysis 20

   2.12. Software used 20

3. RESULTS 21

   3.1. Ecological model results 21

   3.2. Economic model results 21

   3.4. Budget Impact Analysis 30

4. REFERENCES 37

1. STUDY DESIGN AND MODEL STRUCTURE

1.1. Study design

We constructed an age-stratified decision tree economic model to assess the global impact of preventable burden of pneumococcal disease in children under five years of age over 30 years period time-horizon, as well as the cost-effectiveness of introducing routine PCV-13 vaccination in 180 UN member-states - where 52 countries have yet to introduce PCV-13 using standard decision analysis methods.[1] In our analysis, we included a total of 4·55 billion children under five. The decision tree compared two strategies: vaccination or no vaccination. The vaccination scenario includes both the ecological impact of vaccination and the economic model, described below. The model assesses risk of various clinical presentations of pneumococcal disease -meningitis, pneumonia, non-pneumonia non-meningitis (NPNM) IPD and AOM- in both vaccinated and unvaccinated children. Each disease was associated with a cost and health utility loss. Disease may result in the affected individual being admitted to hospital, treated at a health centre or simply not seeking care. Finally, the long-term resulting health outcomes are making a complete recovery, recovering with sequelae and dying. In the main article the Figure 1 shows the age-stratified decision tree economic model developed to represent disease outcomes and associated health states for vaccinated and unvaccinated populations in our model. The same structure is repeated for every year of age between 0 to 5 years. The 'no vaccination' node has the same branches as the vaccination node.

1.2. Ecological vaccine impact

In order to account for direct and indirect vaccine effects, we adapted a recently developed ecological model that is highly predictive of the impact of PCV-7 on invasive pneumococcal disease (IPD) in children living in high income countries [2]. The term "ecological" is used here in the epidemiological rather than biological sense, where the unit of analysis is the population rather than the individual.The model was adapted to account for net effect on disease of potential reductions in vaccine-type IPD in both vaccinated and unvaccinated groups- providing evidence of strong herd effects- and for the expected increases in disease caused by serotypes not targeted by vaccination - and hence able to occupy the ecological niche opened by the reduction in carriage due to vaccine serotypes.[3,4] It simplifies long-term impact predictions, including serotype replacement and herd protection, from more elaborate SIS-type dynamic transmission models into a single predictive equation by making a number of assumptions: (a) as a result of PCV use vaccine serotypes will eventually be eliminated, (b) eliminated serotypes will be fully replaced in carriage by non-vaccine serotypes and (c) that the propensity of the non-vaccine type group to cause invasive disease if carried remains the same in the post PCV era. Then the predicted incidence rate ratio is:

$$IRR = \frac{c+1}{d+1}$$

, where c and d are the odds of VT carriage and IPD respectively. Given the assumption of vaccine serotype elimination, model predictions should be treated as estimates of the maximum reduction in IPD that can be achieved through vaccination, rather than necessarily predictions of vaccine impact; in particular in those settings with low vaccine coverage and/or intense transmission vaccine serotypes may not eliminate vaccine serotypes. Hence we present a sensitivity analysis assuming that reduction in VT carriage is only 65% as observed in Kilifi, Kenya, and hence PCV impact is only 65% of our model's base-case prediction.[5]

It is also assumed that the proportionate reduction in disease over time following vaccination follows the same time course as that observed in a multi-country review of post-vaccination data,[3]

We focus on vaccine impact on children under five years of age by conservatively assuming that there is no impact of PCV on individuals older than five years. Currently, data are limited on the serotype distribution of pneumococcal disease in adults; some studies in high income countries suggest that reductions in vaccine-type disease are mitigated by increases in non-vaccine type disease to a greater extent than in children.[6,7] Furthermore, we assumed that the impact of PCV-13 on non-invasive pneumococcal pneumonia (nIPD) is similar to that on IPD.

Our quasi-dynamic method is less likely to be appropriate to predict PCV impact on otitis media because of the non-linear effects of preventing recurrent episodes.[8] Hence, we assume that PCV-13 vaccination would reduce all cause AOM incidence by 19%, as estimated in the COMPAS trial,[9] regardless of the underlying serotype distribution of disease.

We use the serotype coverage amongst pediatric IPD isolates for PCV-13 before routine use of PCVs, stratified by UN regions, that has been reported earlier in a globl meta-analysis.[10] A corresponding review and global meta-analysis on the regional serotype coverage among healthy children carrying pneumococcus before routine use of PCV was separately commissioned by WHO, and preliminary unpublished results have been used to inform this work[11].

The ecological model was used to predict the regional proportion of IPD and non-bacteraemic pneumococcal disease that is preventable by routine PCV-13 use. The number of each disease cases were then generated by multiplying the full posterior of the IRR estimates with the population size under five years of age and the incidence rates of the disease. In other words, it is the same as multiplying the full posterior of IRR with the number of cases of the diseases in children under five in the absence of vaccination. These IRRs were then used in the economic model via the number of each disease cases.

<u>Equations of vaccine introduction on IPD, nIPD and AOM cases and deaths:</u>

$$\text{Cases\_IPD}_{i,j,t} = \text{Popsize}_{i,yr} * \text{Incidence\_IPD}_{j} * \text{IRR}_{i,j,t}$$

$$\text{Death\_IPD}_{i,j,t} = \text{CasesIPD}_{i,j,t} * \text{Cfr\_IPD}_{j}$$

$$\text{Cases\_nIPD}_{i,j,t} = \text{Popsize}_{i,yr} * \text{Incidence\_nIPD}_{j} * \text{IRR}_{i,j,t}$$

$$\text{Death\_nIPD}_{i,j,t} = \text{Cases\_nIPD}_{i,j,t} * \text{Cfr\_nIPD}_{j}$$

$$\text{Cases\_AOM}_{i,j,t} = \text{Popsize}_{i,yr} * \text{Incidence\_AOM}_{i.j} * \text{vaccine.efficacy.AOM} * \text{DPT.coverage}$$

where $\text{Cases\_IPD}_{i,j,t}$, $\text{Cases\_nIPD}_{i,j,t}$, $\text{Cases\_AOM}_{i,j,t}$ is the number of IPD, nIPD and AOM cases respectively for age i, j bootstrap sample, t time after vaccination, $\text{Popsize}_{i,t}$ is the population size at age i and yr year (from UN population projection where year 2015 correspond to the start of vaccination (t=0)), Incidence_IPD_{j} is the incidence of Meningitis, NPNM IPD, and Pneumonia obtained from O'Brien et al. (2009) that estimated burden of disease caused by Streptococcus pneumonia in children, globally and for each WHO region, $\text{Incidence\_nIPD}_{j}$ is the incidence of

pneumonia (O'Brien) multiplied by the proportion of pneumococcal pneumonia that is non-bacteraemic based on meta-analysis (Said MA et al. (2013)), $\mathrm{IRR}_{i,j,t}$ is the incidence rate ratio of post to pre-vaccination pneumococcal disease described using the equation IRR=(c+1)/(d+1), where c and d are the odds of vaccine type carriage and IPD respectively, $\mathrm{Death\_IPD}_{i,j,t}$ *and* $\mathrm{Death\_nIPD}_{i,j,t}$ is the number of death from IPD and nIPD for age i, j bootstrap sample, t time after vaccination, CfrIPD_j *and* $\mathrm{Cfr\_nIPD}_j$ is the case-fatality risks (CFRs) for meningitis, NPNM IPD and pneumonia from O'Brien et al. (2009) Vaccine efficacy against AOM was assumed to be 19% (COMPAS Trial - Tregnaghi et al. 2014) and DPT coverage is obtained from WHO/UNICEF.

$$\mathrm{TotalCases\_IPD}_j = \sum_{i,t} \mathrm{Cases\_IPD}_{i,j,t}$$
$$\mathrm{TotalCases\_nIPD}_j = \sum_{i,t} \mathrm{Cases\_nIPD}_{i,j,t}$$
$$\mathrm{TotalCases\_AOM}_j = \sum_{i,t} \mathrm{Cases\_AOM}_{i,j,t}$$
$$\mathrm{TotalDeath\_IPD}_j = \sum_{i,t} \mathrm{Death\_IPD}_{i,j,t}$$
$$\mathrm{TotalDeath\_nIPD}_j = \sum_{i,t} \mathrm{Death\_nIPD}_{i,j,t}$$

where $\mathrm{TotalCasesIPD}_j$, $\mathrm{TotalCases\_nIPD}_j$, $\mathrm{TotalCases\_AOM}_j$ and $\mathrm{TotalDeathIPD}_j$, $\mathrm{TotalDeath\_nIPD}_j$ are the total cases and death across 30 years for children under 5 years in j bootstrap respectively.

1.3. Economic model

The economic model was developed to assess the economic implications of different clinical presentations of pneumococcal disease, at different ages, in vaccinated and unvaccinated individuals, and the cost effectiveness of introducing PCV-13 globally.

The analysis was performed to reflect the societal perspective. The health system costs included the cost of healthcare provision, and the vaccination program of PCV-13 vaccination (costs related to both procurement and administration of the vaccines). Societal costs additionally incorporated out-of-pocket expenses and productivity losses borne by households as a result of the disease. All unit prices were converted to 2015 international dollars (I$) as described in the sections below.[12,13] We assumed no changes in other technologies that might affect pneumococcal disease incidence and related outcomes. Future costs and outcomes were discounted at 3% per annum as disaggregated on the results tables. Discounting allows for comparison of costs and benefits across different time periods, by weighting future gains and losses less heavily than those in the present to account for time value. In the presentation of results, all model outputs were rounded to three significant figures.

2. PARAMETERS

For the ecological model, only the regional serotype distributions among healthy children and those with IPD were used. In particular, only the economic model takes into account the following details on demographics, vaccine coverage and vaccination cost, disease incidence and case fatality ratios, healthcare utilization and related costs, health care seeking behavior, related societal costs and Utility values.

We collected individual data for 180 UN countries from six UN regions: Africa (53 countries), Asia (45 countries), Europe (39 countries), LAC (31 countries), North America (two countries) and Oceania (10 countries). Several countries were excluded due to insufficient data on key demographic and economic parameters, namely age-stratified population sizes and GDP PPP. Excluded countries only account for approximately 1% of the global population altogether. See Table 1 for the list of countries. Other parameters with missing values were imputed from regional estimates, based on UN regions or Income-classifications. Regional estimates used for the model parameters, were derived from the weighted averages of countries with available data of the corresponding region where the weights are specified by various criteria, see Table 2.

We also stratified the results by grouping countries according to their Gavi AMC eligibility and/or PCV vaccination introduction (including planned introduction) status.[14,15] We grouped countries that have not implemented vaccination, but have already planned introduction of the vaccine into their national immunization schedules, together with those that have already introduced PCV-13. The groupings included:

- All countries that are pending vaccine introduction;
- All Gavi AMC eligible countries;
- All Gavi AMC eligible countries that have already introduced the vaccine; and,
- All Gavi AMC countries that are pending for vaccine introduction

Table 1. Countries* in UN region

AFRICA (n=53)**	ASIA (n=45)**	EUROPE (n=39)	LAC (n=31)	North America (n=2)	OCEANIA (n=10)
Algeria	Afghanistan	Albania	Antigua and Barbuda	Canada	Australia
Angola	Armenia	Austria	Argentina	United States of America	Fiji
Benin	Azerbaijan	Belarus	Bahamas		Kiribati
Botswana	Bahrain	Belgium	Barbados		Micronesia (Federated States of)
Burkina Faso	Bangladesh	Bosnia and Herzegovina	Belize		
Burundi	Bhutan	Bulgaria	Bolivia - Plurinational State of		New Zealand

AFRICA (n=53)**	ASIA (n=45)**	EUROPE (n=39)	LAC (n=31)	North America (n=2)	OCEANIA (n=10)
Cameroon	Brunei Darussalam	Croatia	Brazil		Papua New Guinea
Cape Verde	Cambodia	Czech Republic	Chile		Samoa
Central African Republic	China	Denmark	Colombia		Solomon Islands
Cape Verde	Cyprus	Estonia	Costa Rica		Tonga
Chad	Democratic People's Republic of Korea	Finland	Cuba		Vanuatu
Comoros	Georgia	France	Dominican Republic		
Congo	India	Germany	Ecuador		
Côte d'Ivoire	Indonesia	Greece	El Salvador		
Democratic Republic of the Congo	Iran - Islamic Republic of	Hungary	Grenada		
Djibouti	Iraq	Iceland	Guatemala		
Egypt	Israel	Ireland	Guyana		
Equatorial Guinea	Japan	Italy	Haiti		
Eritrea Ethiopia	Jordan	Latvia	Honduras		
Eritrea Ethiopia Gabon	Kazakhstan	Lithuania	Jamaica		
Eritrea	Kuwait	Luxembourg	Mexico		
Ethiopia Gabon Gambia	Kyrgyzstan	Malta	Nicaragua		
Ghana	Lao People's Democratic Republic	Montenegro	Panama		
Guinea	Lebanon	Netherlands	Paraguay		
Guinea- Bissau	Malaysia	Norway	Peru		
Kenya	Maldives	Poland	Saint Lucia		
Lesotho	Mongolia	Portugal	Saint Vincent and the Grenadines		
Liberia	Myanmar	Republic of Moldova	Suriname		
Libya		Romania	Trinidad and Tobago		
Madagascar	Nepal	Russian Federation	Uruguay		

**AFRICA (n=53)****	**ASIA (n=45)****	**EUROPE (n=39)**	**LAC (n=31)**	**North America (n=2)**	**OCEANIA (n=10)**
Malawi	Oman	Serbia	Venezuela – Bolivarian Republic of		
Mali	Pakistan	Slovakia			
Mauritania	Philippines	Slovenia			
Mauritius	Qatar	Spain			
Morocco	Republic of Korea	Sweden			
Mozambique	Saudi Arabia	Switzerland			
Namibia	Singapore	The Former Yugoslav Republic of Macedonia			
Niger	Sri Lanka	Ukraine			
Nigeria	Tajikistan	United Kingdom			
Rwanda	Thailand				
Sao Tome and Principe	Timor-Leste				
Senegal	Turkey				
Seychelles	Turkmenistan				
Sierra Leone	United Arab Emirates				
South Africa	Uzbekistan				
South Sudan	Viet Nam				
Sudan	Yemen				
Swaziland					
Togo					
Tunisia					
Uganda					
United Republic of Tanzania					
Zambia					
Zimbabwe					

* 17 countries/areas/territories in UN regions were dropped as they were not in WHO regions: China - Hong Kong SAR, Mayotte, Aruba, French Guiana, Martinique, Channel Islands, State of Palestine, Western Sahara, French Polynesia, Guam, New Caledonia, Réunion, China - Macao SAR, Curaçao, Guadeloupe, Puerto Rico, United States Virgin Islands. ** Insufficient data was available for three countries: Democratic People's Republic of Korea (Asia), Somalia (Africa) and Syrian Arab Republic (Asia).

Table 2. Base case parameters used in the model

Parameter	North America	Africa	Asia	Europe	LAC	Oceania	Source
Vaccine characteristics	Assumption when the corresponding countries do not have immunization program						
Vaccine schedule	3+1	3+0	3+0	2+1	2+1	3+0	WHO and Whitney CG et al. 2014
PCV-10/13 effectiveness against all-cause AOM	19%	19%	19%	19%	19%	19%	COMPAS trial
Coverage (in targeted population)							
... dose one*	96.9%	86.2%	91.7%	95.4%	96.1%	91.9%	WHO/UNICEF
... dose two*	95.8%	82.3%	90.1%	93.6%	93.0%	87.2%	Assumption average (dose one and three)
... dose three*	94.6%	78.4%	88.4%	91.8%	89.9%	82.6%	WHO/UNICEF
... dose four	90.1%						CDC MMWR
Costs (USD 2015)							
Vaccination in USD (before adjustment to International Dollars)							
... purchase (PCV-13)**	120.39	3.66	15.68	120.39	15.68	15.68	CDC/Gavi AMC/PAHO
... freight (PCV-13)	7.22	0.22	0.94	7.22	0.94	0.94	Assumption
... buffer stock	25%	25%	25%	25%	25%	25%	WHO guidelines
... Cold chain(per dose)	0.405	0.405	0.405	0.405	0.405	0.405	WHO/UNICEF Guidelines and Usuf E. 2014
Hospitalization (I$)							
…meningitis	20498.07	1175.82	3156.01	9849.55	5392.03	6334.78	Portnoy et al (LMIC) and other PCV CEAs
…pneumonia	3792·66	111·15	347·41	1982·66	468·62	1910·30	WHO CHOICE and Zhang et al
…invasive NPNM	7464·12	305·28	1732·40	5883·11	4303·68	5057·08	Various PCV CEAs
Health centre consultation (I$)							
…acute otitis media	154·35	14·07	50·30	93·30	44·88	32·64	Various PCV CEAs
Productivity costs							
…female labour force participation rate***	67·0%	57·2%	50·3%	66·5%	57·6%	69·9%	ILO
…Gross domestic product (PPP) per capita[3]	54,653·15	5,210·55	12,058·82	32,353·03	15,280·18	34,542·08	World Bank/IMF
Out of pocket costs							
… % expenditures on total healthcare	11·3%	43·2%	48·8%	25·7%	34·0%	14·7%	WHO Global Health Expenditure Database

Parameter	North America	Africa	Asia	Europe	LAC	Oceania	Source
... meningitis	2598·33	1024·60	2377·03	4466·30	3375·23	1360·27	WHO Global Health Expenditure Database
... pneumonia	479·84	107·94	249·12	582·55	312·73	413·69	WHO Global Health Expenditure Database
... invasive NPNM	947·32	346·64	1737·68	2163·15	2519·25	1000·92	WHO Global Health Expenditure Database
... acute otitis media	19·54	16·49	41·71	30·18	26·23	6·91	WHO Global Health Expenditure Database
Proportion of patients who seek care							
… Acute otitis media (health care centre)**	85·1%	52·14%	63·09%	85·1%	63·09%	63·09%	UNICEF MICS Care-seeking for fever
… Non-invasive pneumonia (hospital)**	100%	49·6%	66·7%	100%	66·7%	66·7%	UNICEF MICS care-seeking for pneumonia
Risk of Pneumonia sequelae	34·7%	34·7%	34·7%	34·7%	34·7%	34·7%	Risk of at least one major sequela in Edmond K, et al 2010
Disability weights							
… Meningitis (per episode)	0·62	0·62	0·62	0·62	0·62	0·62	“Meningitis, *S. pneumoniae*” in GBD- Mathers et al. 2006
… Pneumonia (per episode)	0·28	0·28	0·28	0·28	0·28	0·28	“Neonatal pneumonia” in GBD- Mathers et al. 2006
… NPNM (per episode)	0·15	0·15	0·15	0·15	0·15	0·15	“Meningococcaemia without meningitis” in GBD - Mathers et al. 2006
… Otitis media (per episode)	0·02	0·02	0·02	0·02	0·02	0·02	“Otitis media” in GBD - Mathers 2006
… Meningitis sequelae (per episode)	0·06	0·06	0·06	0·06	0·06	0·06	Meningitis sequelae in GBD- Mathers et al. 2006

Note: The variables in the table are solely used for comparison between the regions (except vaccine characteristics, buffer stock percentage, cold chain cost, risk of pneumonia sequelae, and disability weights)

* Regional estimates are weighted by the size of population under 1 year old of countries of the corresponding regions.
** Parameters depend on the income classification, rather than the UN regions. The regional estimates here reflect countries with most prevalent income class (Africa – low income, Asia – middle income, Oceania – middle income, LAC – middle income, Europe – high income, and North America – high income). Vaccine prices also depend on whether the country is subsidized by Gavi or not, see methodology for more detail on this.
*** Regional estimates are weighted by the size of population between 15 to 64 years old of countries of the corresponding regions.

2.1. Demographics

Age-specific population figures for 180 countries were obtained from United Nations Population Division estimates.[16] Mortality rates were also calculated from UN population data.[17] Life expectancies at each five-year age interval were estimated from the mortality rates in each country using the WHO life table method. For the 1-4 year-old range, the five-year life expectancy was attributed to three-year-old children, making this the age of highest life expectancy consistent with higher mortality in children younger than three years old. In more detail. We used population tables of the under-5 from United Nations Population Division from 2015 to 2045 as inputs to our model in the calculation of the number of cases of IPD, nIPD and AOM. For example, the number of cases of IPD after vaccine introduction was calculated based on the population size, the incidence of IPD and IRR.

$$\mathrm{Cases\_IPD}_{i,j,t} = \mathrm{Popsize}_{i,yr} * \mathrm{Incidence\_IPD}_{j} * \mathrm{IRR}_{i,j,t}$$

for age i, j bootstrap sample, t time after vaccination, $\mathrm{Popsize}_{i,t}$ is the population size at age i and yr year (from UN population projection where year 2015 correspond to the start of vaccination (t=0))

Population size for the under-5 from year 2015 (t=0th year of vaccination) to 2045 (t=30th year of vaccination) was based on country specific fertility under the medium variant assumption. Table S5 below the fertility by region.

TABLE S.5. TOTAL FERTILITY BY COUNTRY AND REGION FOR SELECTED PERIODS (MEDIUM VARIANT)

	Total fertility (live births per woman)						
Region, country or area	*1975-1980*	*1990-1995*	*2005-2010*	*2010-2015*	*2015-2020*	*2025-2030*	*2045-2050*
**World**	3.87	3.02	2.57	2.52	2.47	2.39	2.24
**Africa**	6.64	5.72	4.89	4.72	4.43	3.90	3.09
**Asia**	4.10	2.92	2.30	2.20	2.15	2.06	1.90
**Europe**	1.98	1.57	1.55	1.60	1.62	1.69	1.78
**Latin America and the Caribbean**	4.48	3.06	2.26	2.14	2.04	1.89	1.77
**Northern America**	1.77	2.00	2.01	1.85	1.86	1.87	1.89
**Oceania**	2.73	2.49	2.53	2.41	2.34	2.23	2.06

Source: https://esa.un.org/unpd/wpp/publications/Files/WPP2017_KeyFindings.pdf

2.2. Meningitis, Pneumonia, NPNM and Acute Otitis Media - Incidence and Case-Fatality Risks:

Meningitis, NPNM IPD, and Pneumonia - The incidence and case-fatality risks (CFRs) for meningitis, NPNM IPD and pneumonia were obtained from a study by O'Brien et al. (2009) that estimated burden of disease caused by *Streptococcus pneumonia* in children, globally and for each WHO region.[18] Since this study provided incidence and CFR estimates for the region of Americas as a whole, these estimates were applied to both North America and LAC regions in the current analysis.

Uncertainty distributions for total incidence and case-fatality risk for meningitis, NPNM IPD and pneumonia in each region were assumed to follow negative binomial distribution and beta distribution respectively. The parameters of the distribution are based on the confidence intervals reported in the study by O'Brien et al.[18] These parameters are generated by finding the estimates that yield the closest intervals as reported by the study. Region-specific incidence estimates and CFR estimates used in this analysis are presented in the Figure 1 and Figure 2 in this document (next pages), respectively.

In order to differentiate non-invasive pneumococcal pneumonia (nIPD) and invasive pneumococcal pneumonia (pneumonia IPD), we assumed that the proportion of pneumococcal pneumonia that is bacteraemic was 24.8% based on meta-analyses estimating the burden of pneumococcal pneumonia in adults.[19] The proportion of bacteraemic pneumonia in adults had to be assumed similar to children under 5 due to the lack of available literature. The data from a vaccine trial in Gambia, on children under the age of one, reports the proportion of bacteraemic pneumonia from 2·2% to 50·9% and 24·8% is well within the 95% confidence bound used.[20] The CFR for invasive pneumonia was obtained from O'Brien et al;[18] while the CFR for nIPD was, conservatively, assumed to be zero due to lower severity of illness.

Acute otitis media – Uncertainty distributions for AOM total incidence were assumed to follow negative binomial as well. The figures were obtained from a study by Monasta et al. that estimated global burden of disease for otitis media based on a systematic review of published studies for different WHO region.[21] Age-dependent regional incidence rates were applied to individual countries to obtain number of cases of AOM for each bootstrap.

2.3. Utilities

Disability-Adjusted Life Years (DALYs) incurred due to non-fatal pneumococcal meningitis, pneumonia, NPNM and otitis media were respectively obtained from disability weights per episode for meningitis due to *S. pneumoniae*, neonatal pneumonia, meningococcemia without meningitis and otitis media in the 2000 update to the WHO's Global Burden of Disease. [22] For our sensitivity analyses we used the 2015 update.[23] The risk of major sequelae of pneumococcal meningitis was obtained from a global meta-analysis.[24] The single risk value obtained was then multiplied by the corresponding DALYs incurred to obtain the overall DALYs incurred for meningitis sequelae. All disability weights can be seen in Table 3.

Table 3. DALYs incurred per condition

	**DALYs** incurred (2000)	**DALYs** incurred Sensitivity Scenario (2015)
Meningitis	0·62	0·133 (0·088-0·19)
Meningitis sequelae	0·06	
Pneumonia	0·28	0·133 (0·088-0·19)
Non-invasive pneumonia	0·28 (assumed)	0·051 (0·032-0·074)
NMNP IPD	0·15	0·133 (0·088-0·19)
Acute otitis media	0·02	0·013 (0·007-0·024)

Figure 1. Incidence rates of pneumococcal disease, excluding acute otitis media

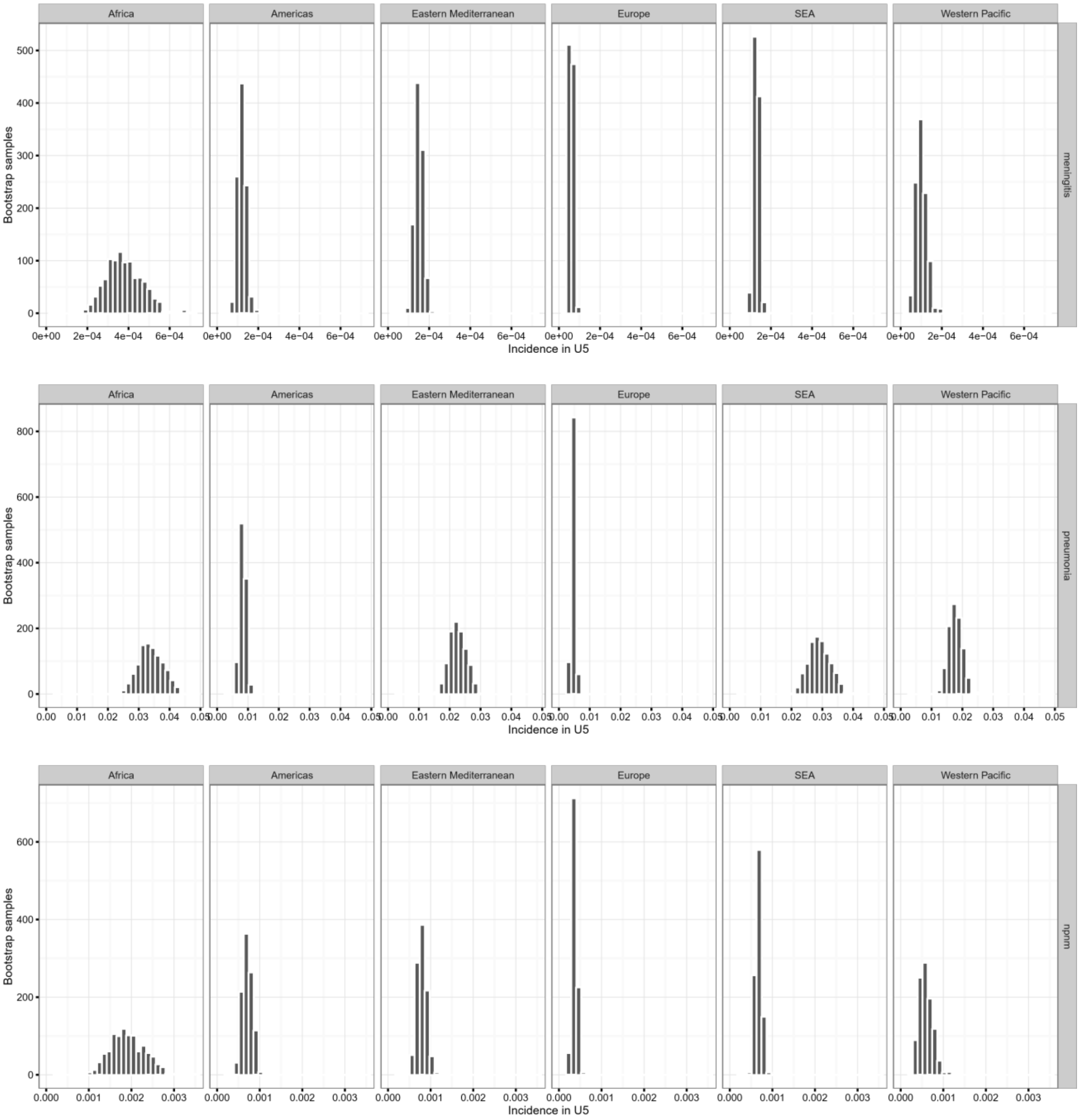

The source for Figure 1 is “O'Brien KL et al. (2009). Burden of disease caused by Streptococcus pneumoniae in children younger than five years: global estimates. Lancet; 374(9693): 893-902”.

Figure 2. CFRs for Meningitis, Pneumonia IPD, and NMNP IPD

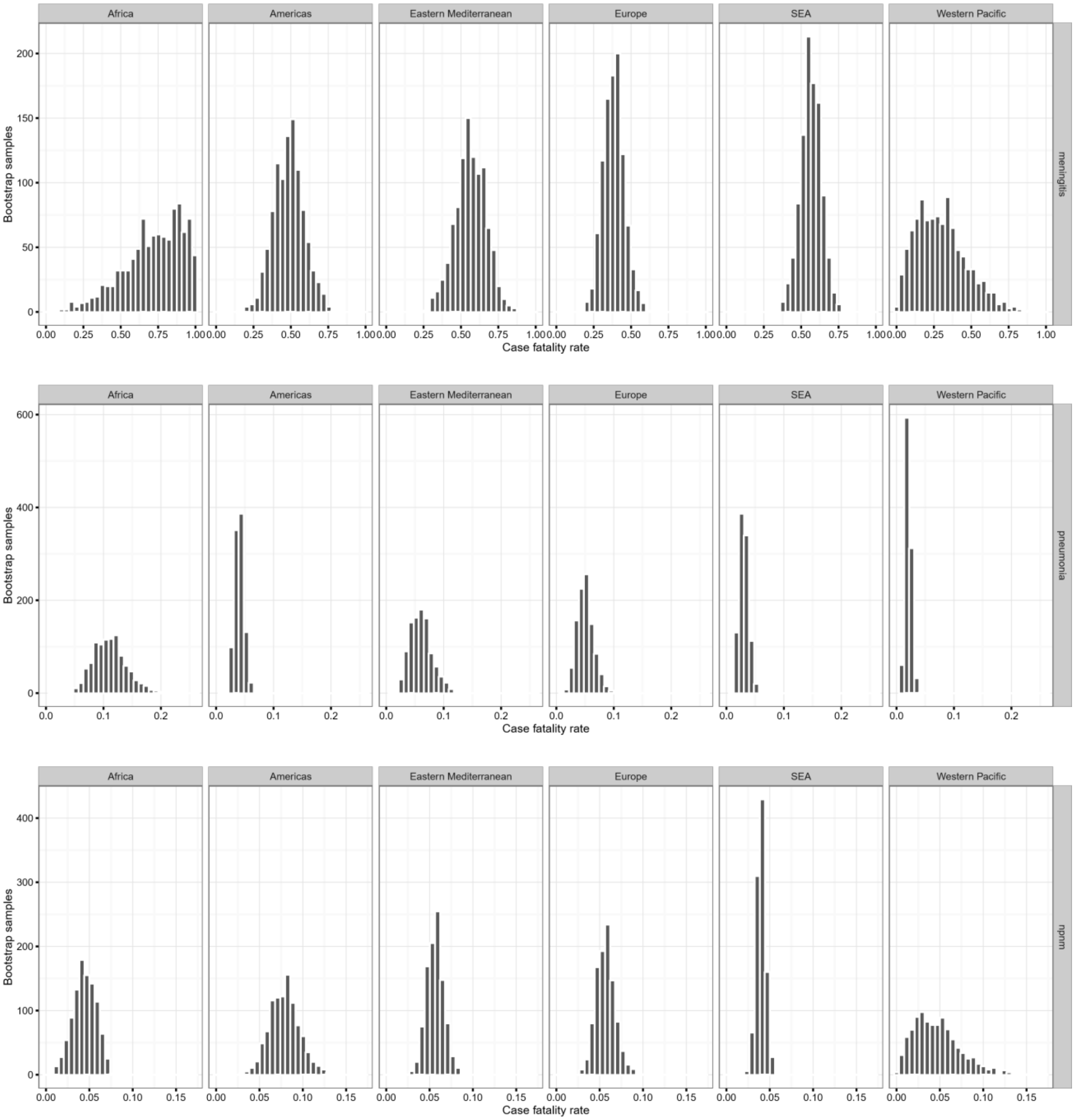

The source for Figures 2 is "O'Brien KL et al. (2009). Burden of disease caused by Streptococcus pneumoniae in children younger than five years: global estimates. Lancet; 374(9693): 893-902".

2.4. Vaccination schedules and coverage

Currently, different schedules for PCV vaccination are used across the world:

- Three primary doses at 6, 10 and 14 weeks followed by a booster at least 6 months later (3+1),
- Three primary doses at 6, 10 and 14 weeks only (3+0),
- Two primary doses at 6 and 14 weeks followed by a booster at least 6 months later (2+1).

In this global analysis, the exact reported individual immunization schedules of 128 countries were used. [15] For countries without reported national immunization schedules, we assumed regional schedules of 2+1 for Europe and LAC, and 3+0 for Africa, Asia and Oceania, as recommended by WHO. [25,26]

Vaccine coverage was assumed to be equal to diphtheria-tetanus-pertussis (DTP) coverage. DTP3 coverage values were obtained for all countries from the WHO/UNICEF Joint Reporting System.[27] All modeled schedules and coverage levels were assumed sufficient to lead to full vaccine serotype elimination in IPD in the ecological impact model. The second dose coverage was assumed to be the average of coverage for the first (DTP1) and third (DTP3) dose. For countries with a 3+1 schedule, it was assumed that coverage of the fourth dose vs. the third dose maintains the same ratio as PCV ≥4 doses vs PCV ≥3 doses coverage. As these countries have similar DTP coverage to the United States, fourth does coverage was based on the USA 2015 National Immunization Survey coverage data.[28] The resulting coverage of the fourth dose was calculated as 90·1% of the third dose.

2.5. Vaccination program cost

Vaccination program costs included vaccine purchase costs, vaccine introduction cost and vaccine administration costs. The descriptions for vaccine purchase costs and vaccine administration costs listed below are per dose, purchased and delivered respectively. Therefore, we take into account birth cohort size, Diphtheria-Tetanus-Pertussis (DTP) vaccine coverage as a proxy for PCV coverage as described above, and the doses per child needed according to individual countries immunization schedules to compute total cost. A summary of the regional parameters (per dose) is provided in Table 2 above.

Vaccine purchase costs–Three levels of vaccine prices were used Gavi AMC eligibility and the country's income group, as per World Bank classification of 2015 fiscal year. [14,29] For countries considered high-income, a price of USD 120·39 per dose was used for PCV-13 based on the US Centers for Disease Control and prevention (CDC) vaccine price list.[30] For countries considered middle-income, the price of PCV-13 was assumed to be USD 15·68 per dose based on the latest prices available to the Pan-American Health Organization (WHO PAHO) revolving fund.[31] For the Gavi eligible and low-income countries, a price tag of USD 3·05 per dose for PCV-13 was used, based on a recent PCV price reduction similarly to the price paid to the vaccine suppliers by Gavi for eligible countries. [14,32] In addition, for these, Gavi countries, a 20% increase price was added to the Gavi price to account for the Advanced Market Commitment (AMC) payments to manufacturers.[33] The number of doses given was calculated taking into account birth cohort and DTP coverage and the doses per child needed according to individual countries immunization schedules as stated above. In order to calculate the total number of doses purchased, we added a buffer stock of 25% of first birth cohort,[34] that was assumed to be maintained constant at all time, and a 5% wastage rate of the number of doses to be given.[35] Wastage was assumed to increase

overall costs without reducing the number of children vaccinated. An additional 6% of the vaccine price was added to account for freight costs.

Vaccine introduction activities and cold chain – To provide illustrative costs of vaccine introduction a vaccine introduction grant and cold chain needs were calculated.

The introduction grant was calculated to partially reflect the cost of pre-introduction activities such as training and education campaigns. Per each children on the initial birth cohort I$ 0·60 was added, or a I$ 100,000 lump sum, whichever was higher, in a similar fashion to Gavi policies.[36]

Cold chain costs were estimated based on total volume calculations as per WHO guidelines,[37] assuming a constant cost of USD 0·406 per dose procured based on a study of incremental costs for vaccine introduction and UNICEF vaccine presentation volumes.[38,39] This was not adjusted for country income level since most components of the cold chain are non-tradeable goods.[40]

Vaccine administration costs - Related injection supplies costs were also calculated and included in the model. AD syringes at USD 0·04 and safety boxes at USD 0·005 per dose needed were calculated and the wastage rates, as per vaccines, were assumed.[41] All the vaccine, AD syringe, Safety Box, and cold chain costs were converted to International dollars (I$) using the World Bank and International Monetary Fund price level ratios of GDP per capita (PPP) to market exchange rate in 2015.[12,13]

To account for the opportunity cost of healthcare staff time to vaccinate the children, the cost of five minutes of a nurse's time per each dose administered was assumed. Professional nurse salaries for 54 countries were obtained from the International Labor Organizations' Occupational Wages of the World database.[42] The salaries were converted into I$ using both exchange rates and PPP conversion rates.[12,13,43] The salaries were then adjusted to year 2015 by multiplying the I$ values with the ratio between GDP per capita (PPP) in year 2015 and in the corresponding years.[44] A linear regression model was fit to the data using GDP per capita (PPP) in 2015 as explanatory variables. The correlation was highly significant with an adjusted R^2 of 64·9%. Countries with non-available salaries data are then imputed with this regression model.

2.6. Healthcare utilization

In order to estimate health care utilization rates, different approaches for invasive and non-invasive pneumococcal disease were adopted.

For invasive pneumococcal disease (IPD), due to greater severity, we assumed that 100% of cases seek inpatient hospitalization.

For non-invasive pneumococcal disease (nIPD), UNICEF data on health care seeking behavior was used. To determine the percentage of children under age five, with pneumonia symptoms, who are taken to hospital data on care-seeking behavior for pneumonia was used.[45] For AOM, we assume that these cases are treated in health care centers due to the milder nature of the condition. Care seeking behavior for fever for children under five was used to inform the proportion of these cases who visited health care centers.[46]

In case of countries with missing data, we set the percentage to be the income-level related values as per the region where the countries are located. The regional values are based on the weighted

average of the proportion from other countries of the same income and region with available data. The weights used are derived from the size of population under five.

For high income countries, where there are no data at from UNICEF, we assume that 100% of children with non-invasive pneumonia seek medical care and that 85·1% of children with AOM. The latter value is based on studies from high income countries estimating that approximately 70-100% of children with acute otitis media seek medical care.[47] In Table 2, the regional proportions of cases of non-invasive pneumococcal disease who seek care are summarized. We use these regional estimates to impute the countries with missing proportion of cases.

In the sensitivity analysis that varies costs using WHO-CHOICE costs,[48] the lengths of stay of each inpatients disease episode were taken into account. The lengths of stay were calculated using etimates from various sources. For meningitis an average length of stay of nine days was used, based on peer reviewed evidence in paediatric cases by Mongelluzo et al.[49] The length of stay for pneumonia was assumed as 3·9 days based on average length of stay of children for pneumonia in the United States.[50] For NPNM, 6·32 days was assumed, based on the average length of stay for septicemia in the United States for adult (8·9 days) and the previously estimated proportion of duration of pediatric stays for all causes (29% shorter).[51,52]

2.7. Healthcare costs

The costs of treating a patient with pneumococcal disease (meningitis, pneumonia, or NPNM invasive pneumococcal disease) were predicted using best fit model generated from data extracted from the literature. PCV cost-effectiveness analysis (CEA) studies were found through a ciritical literature review and/or included in four main systematic reviews; from those two costing studies and 27 CEA studies were selected and used.[53–85] The costs were extracted and converted into International dollars at corresponding years the studies reported and then adjusted to 2015 using the ratio between GDP per capita PPP in year 2015 and those corresponding years.

Meningitis

Hospitalisation costs for Meningitis in low-middle income countries (LMIC) were extracted from a study by Portnoy et al that predicted costs for all LMICs by conducting a systematic review and extrapolating data to set up a database on cost of care for childhood meningitis.[80] For high income countries (HIC) we extracted data from thirteen PCV CEA studies (N = 15 countries).[57,59,63,66,67,70,71,73,75,78,83–85] As the costs were not skewed, log transformation was not required. The predictors used were GDP per capita PPP in year 2015 and UN regions indicators. In search for the best fitting model, we used stepwise regression with AIC criterion. The resulting model was:

Cost = -1711·32 + 0·27*GDP per capita PPP + 7658·29 * I(North America = 1)

The adjusted R-squared was 50·8%. Through this model, we predict the costs of all the HIC countries (N = 46 countries), including the 15 countries whose data were used to generate the model.

Pneumonia

Hospitalisation costs for pneumonia were also stratified by LMICs and HICs based on data extracted in study by of Zhang et al. wich conducted a systematic review of studies providing the cost of childhood neumonia and also included unpublished data at that time.[81] In their study the cost for LMICs was extracted from severe pneumonia data which mean is 242·7 USD 2013 (and also I$ 2013). As costs per episode for each country, were not available, we used WHO-CHOICE inpatient costs per day costs per day multiplied by 3·9 days as a proxy for costs per episode.[48] The 3·9 days value was the average length of stay for pneumonia disease based on average length of stay for pneumonia in the United States.[50] The resulting values were converted to International dollars of year 2015 (I$ 2015). To incorporate this information, we used weighted averages, with weights being the population size under five years of age, and the resulting weighted costs were compared with the extracted costs from Zhang et al.[81] We use the resulting value as a multiplicative factor. Finally, we applied this factor to the costs that we have generated. The multiplicative factor for LMIC was 1·18 and for HIC was 1·37;

NPNM

To estimate hospitalisation costs for NPNM, costs from 25 PCV CEA studies were extracted (N = 29 countries).[56–64,66–73,75,77–79,82–85] As the available costs were skewed, we used the logarithm of costs as our outcome. The predictors used were the logarithm of GDP per capita PPP in year 2015 and UN regions indicators. In search for the best fitting model, we used stepwise regression with AIC criterion. The resulting model was:

$$\ln(\text{cost}) = 4{\cdot}33 + 0{\cdot}41*\ln(\text{GDP per capita PPP}) - 0{\cdot}73*I(\text{Asia} = 1) - 2{\cdot}08*I(\text{Africa} = 1)$$

The adjusted R-squared was 65·5%. To transform back to cost, we needed to have a correcting factor. [86] The correcting factor equals to the sum of the expected residuals. In this case, it was 1·108. Therefore, the cost model used was:

$$\text{Cost} = \exp(4{\cdot}33 + 0{\cdot}41*\ln(\text{GDP per capita PPP}) - 0{\cdot}73*I(\text{Asia} = 1) - 2{\cdot}08*I(\text{Africa} = 1))*1{\cdot}108$$

Through this model, we predicted the costs of all the countries, including the 29 countries whose data were used to generate the model.

AOM

Outpatient treatment costs for AOM were extracted from 24 PCV CEA studies (N = 25 countries).[55–57,59–64,66–68,70–75,77–79,83–85] As the resulting costs were skewed, we used the logarithm of costs as our outcome. The predictors used were the logarithm of GDP per capita PPP in year 2015 and UN regions indicators. In search for the best fitting model, we use stepwise regression with AIC criterion. The resulting model was:

$$\ln(\text{cost}) = -5{\cdot}46 + 0{\cdot}94*\ln(\text{GDP per capita PPP}) + 0{\cdot}45*I(\text{Asia} = 1) - 0{\cdot}89*I(\text{Oceania} = 1)$$

The adjusted R-squared was 42·8%. To transform back to cost, we had a correcting factor [86]. The correcting factor equals to the sum of the expected residuals. Here, it was 1·218. Therefore, the cost model wass:

$$\text{Cost} = \exp(-5{\cdot}46 + 0{\cdot}94*\ln(\text{GDP per capita PPP}) + 0{\cdot}45*I(\text{Asia} = 1) – 0{\cdot}89*I(\text{Oceania} = 1))*1{\cdot}218$$

Through this model, we predicedt the costs of all the countries, including the 25 countries whose data were used used to generate the model.

2.8. Societal costs

Societal costs were estimated by identifying productivity losses due to sickness and out-of-pocket expenditure components in healthcare-related costs.

In order to calculate productivity loss a two-step approach was required. Firstly, the average number of days of work lost due to an episode of pneumococcal disease was estimated using the average hospital length of stay for each outcome. For AOM, the number of days of work lost, only for cases involving health care center consultations, was assumed to be one. This was based on the assumption of a half-day diagnostic visit and a half-day follow-up visit being required. Secondly, in each case of children with a disease episode, this value was multiplied by the female labor force participation rate (15 y.o. – 64 y.o.) for the particular country.[87] This was done assuming that mothers were the primary caregiver who would have to take time off work in order to look after the sick children. Finally, each day of work lost was valued based on the Gross Domestic Product per capita (PPP) of each country in 2015 I$.[12,13]

Travel and other associated costs were assumed to be part of OOP expenditures. Out-of-pocket (OOP) expenditures, as the percentage of out-of-pocket expenditures on total health expenditure for individual countries, were obtained from the WHO-Global Health Expenditure Database and regional averages were calculated.[88]

2.9. Incremental Cost-Effectiveness Ratios – ICERs

We estimated the incremental cost-effectiveness ratio (ICER) by comparing the introduction of PCV-13 to no PCV use or [(Cost in PCV-13 arm – Cost in no PCV arm)/(DALYs averted)]. The median of 1000 ICERs bootstraps were calculated on International dollars I$ (2015) and DALYs. We then compared the resulting ICERs with the GDP per capita (PPP) as it has been traditionally used as an indicative threshold to indicate when an intervention is cost-effective.[89] The ICERs were compared with the GDP per capita (PPP) of each region, calculated by summing over population weighted figures for each country. But ,as more recently this threshold has been criticized.[90,91] We also compared the ICERs to the more stringent cost-effectiveness thresholds estimated by Woods et al.[92] to account for the opportunity cost of health expenditure, thus providing more appropriate measure of Cost-Effectiveness Threshold for resource allocation purposes. All the resulting ICERs are presented with a 3% discount rate applied to costs and utility outcomes, as noted earlier.

2.10. Sensitivity analysis

We used Probabilistic sensitivity analysis to explore statistical uncertainty on study results by generating bootstrapping of 1,000 samples where incident cases were modelled using a negative binomial distribution and case-fatality risk follows a beta distribution.[18,20,21] Estimates of the costs, disease outcomes, and ICERs were obtained for each sample, allowing us to generate 95% CIs

with the percentile method. The advantage of this approach is that our confidence intervals of do not rely on the usual normality assumption.

In order to test the robustness of the model, we conducted one-way sensitivity analyses varying key parameters over plausible ranges to assess their effect on the ICERs and the number of deaths. We created high and low disease burden scenarios by varying incidence and case fatality rates within the lower and upper bounds from Obrien et al,[18] and Monasta et al,[21] and the ratio of invasive pneumococcal pneumonia according to the lower and upper bounds of Cutts et al.[20] Vaccine price, and every other non-vaccination cost input, were varied +/-20%. Discount rates, for both, costs and outcomes, were set to 0% or 6%. We changed the model utility values to The Global Burden of Disease 2015 Study DALY weights (See Table 3 above).[23]

We also performed a high coverage subgroup analysis, by excluding 63 countries without national immunization program and/or with DPT3 coverage levels below 70%. We based this subgroup analysis on the assumption that complete elimination of vaccine type *S.pneumoniae* had not been achieved in those countries. We also created a scenario where we used WHO-CHOICE costs,[48] and the lengths of stay as stated above.[49–52]

In an additional scenario we assumed that low and middle-income countries (LMIC) do not achieve complete elimination of VT (Vaccine Type) carriage. We base this scenario on serotype carriage in Kilifi County, Kenya two years after PCV-10 introduction; this setting was chosen because of well-documented carriage from a post-introduction study. [5] This found an adjusted prevalence ratio of 0·36 for children <5 years for vaccine-type pneumococcus, suggesting a 65% reduction instead of full reduction.

2.11. Budget Impact Analysis

We conducted a budget impact analysis to assist on the identification of the financial consequences of vaccination. We estimated the financing flows showing the fiscal impact of vaccines by global / regional / subgroups. The projections of the budget impact analysis aimed to provide guidance while assessing the affordability of PCV introduction.

2.12. Software used

The ecological impact model and the economic model were adapted/developed using Software R Version 3.3.1 (R foundation for Statistical Computing, Vienna, Austria) and Microsoft Excel® (Microsoft corp., Redmond, WA, USA) Software were used.

3. RESULTS

3.1. Ecological model results

The results of the ecological model indicate that PCV-13 could substantially reduce the burden of invasive pneumococcal disease in all regions as seen in the Figure 3 of this document (below). The greatest proportionate reduction in IPD following vaccination is predicted to be in North America. Estimates in Africa, Asia and in particular Oceania include relatively wide uncertainty bounds which take into account both the sparsity of carriage data and the heterogeneity of serotype distribution among healthy carriers in those regions. Here are the 95% confidence intervals:

	**0 years after vaccination**	**>0 years after vaccination**
**North America**	0·43 (0·38-0·47)	0·27 (0·22-0·33)
**Latin America**	0·60 (0·55-0·67)	0·50 (0·43-0·58)
**Africa**	0·74 (0·58-0·89)	0·67 (0·47-0·86)
**Europe**	0·61 (0·54-0·67)	0·50 (0·42-0·59)
**Asia**	0·73 (0·58-0·90)	0·66 (0·47-0·87)
**Oceania**	0·64 (0·42-0·88)	0·55 (0·27-0·85)

Figure 3. Full posterior estimates for the predicted incidence rate ratios for IPD in children under 5 years of age

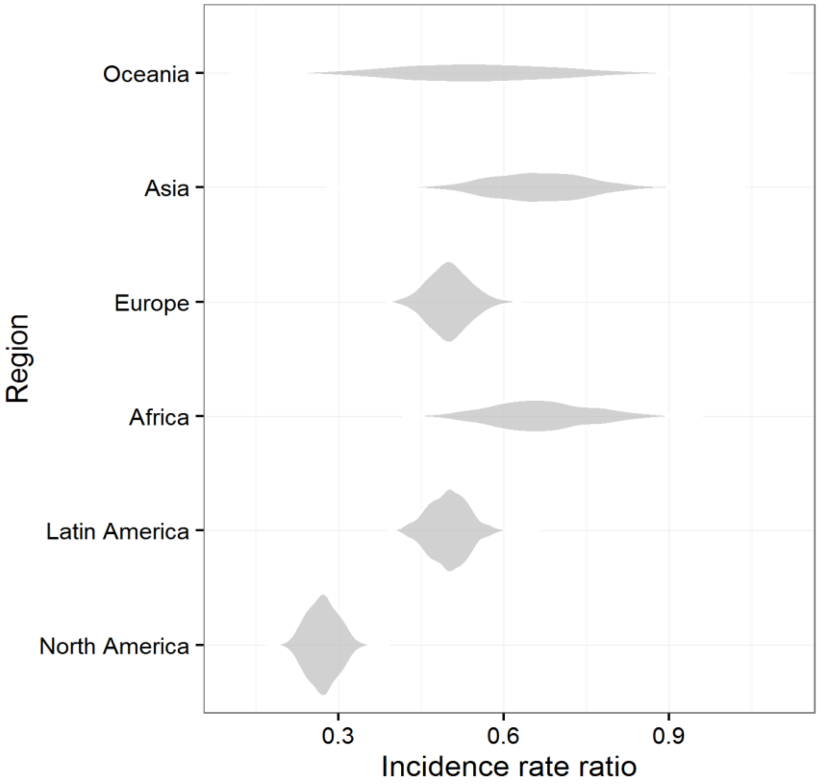

3.2. Economic model results

The result of the economic model indicates that PCV-13 can prevent 12 million deaths, or equivalently 263 deaths per 100,000 children under 5 years old through the 30 years time-horizon globally. The rate of prevented deaths for each country, stratified by countries that have

implemented PCV-13 and countries that do not have existing PCV-13 vaccination programs can be seen in the main article Figure 2. In general, Africa has the highest preventable rate of deaths (548 deaths per 100,000 children) regardless of whether the countries have or have not introduced the vaccine. Overall, if all countries (52 countries with mostly Asia and Africa) that have not implemented the program started to introduce the vaccine, they would reduce 2·7 million deaths, or equivalently 141 deaths per 100,000 children.

Vaccination would be cost-effective at the median ICER of I$ 724·38 per DALY averted. The median ICER of countries, along with each of the country's threshold (both are in logarithmic values) are shown in the main article Figure 3. The colors indicate the region of the country. We used two thresholds to assess cost-effectiveness of the vaccine. One such threshold is obtained from Woods et al data (N = 173, seven countries do not have this data). The other threshold is GDP per capita (PPP) (N = 180). If the ICER for a country is below the specified threshold, then the intervention may be considered cost-effective. This is shown above the diagonal line in the figure. Similarly, if the ICER for a country is above the specified threshold, then the intervention may be considered not cost-effective. This is shown below the diagonal line in the figure. According to our results, if we use Woods et al thresholds, out of 173 countries included, in 143 countries vaccination may be consideredcost-effective (82·7%) and in 11 countries vaccination was cost-saving (6·1%). If we use GDP per capita (PPP) thresholds, out of the 180 countries included, the number of countries in which vaccinatoun may be considered cost-effective would raise to 166 (92·2%). Our results estimate that in three countries namely, Croatia, Poland, and Russian Federation, at current prices and coverage levels, vaccination may not be considered cot-effective while comparing ot any of the two thresholds. The graph also show the incremental cost-effectiveness ratio (ICER) of PCV-13 vaccination vs. no vaccination for each country (x-axis) in comparison with its respective cost-effectiveness threshold (y-axes: A- Woods et al. thresholds, B- 1xGDP per capita (PPP) thresholds). The x-axis represents the cost-effectiveness estimate obtained from our model; the y-axes represent the respective thresholds (both measured in I$ per DALY averted). Points above the line (y=x) are cost-effective. Graphs were presented using a log-log scale, and credible intervals were omitted for clarity.

Tables 4a and 4b present the global and regional base case outcomes of costs, effectiveness, and disease cases associated with no vaccination, vaccine introduction, and their increments respectively. Tables 5a, 5b, and 5c present the subgroup base case outcomes of costs, effectiveness, and disease cases associated with no vaccination, vaccine introduction, and their increments respectively.

In this analysis, following World Health Organization recommendations, future costs and health outcomes were both discounted to their present values (in the year 2015) at a rate of 3% per annum, unless stated otherwise. Both health system (excluding OOP expenditures and productivity losses) and societal (considering the health system costs but including productivity losses and OOP expenditures) perspectives are shown. Except for ICERs, every other result are averaged over 30 years. Figures are reported to three significant figures for visual clarity. Small discrepancies may result due to this rounding.

Table 4a. Global and regional yearly estimates of pneumococcal disease related outcomes without vaccination and PCV-13 vaccinaton in one year

	Global	Africa	Asia	Oceania	Europe	LAC	North America
**Without PCV introduction**							
Health care costs [illegible]	13.7	2.08	7	0.174	1.67	0.771	2.04
Societal costs (undiscounted) (I$	14.3	3.01	8.09	0.129	1.31	0.725	1.07
IPD cases (Millions)	4.78	2.3	2.19	0.0172	0.0565	0.141	0.0673
Non-IPD pneumonia	12	5.53	5.87	0.0453	0.125	0.305	0.146
Acute otitis media	440	237	162	2.49	14.5	15.6	8.51
Deaths (Millions)	1.18	0.852	0.285	0.00139	0.0098	0.0218	0.0104
DALYs lost	47.4	30.6	14.3	0.116	0.684	1.14	0.575
**A. DALYs lost (discounted)**	**31.4**	**20**	**9.73**	**0.0776**	**0.465**	**0.775**	**0.385**
Total costs (undiscounted) (I$, Billions)	28	5.09	15.1	0.304	2.98	1.5	3.11
**B. Total costs (discounted) (I$, Billions)**	**18.9**	**3.34**	**10.3**	**0.203**	**2.02**	**1.02**	**2.08**
Total health system costs* (undiscounted) (I$, Billions)	13.7	2.08	7	0.174	1.67	0.771	2.04
Total health system costs* (discounted) (I$, Billions)	9.26	1.37	4.77	0.117	1.13	0.526	1.36
**PCV-13 vaccination**							
Number of children [illegible]	114	35	58.1	0.561	8.77	6.7	4.42
Vaccination program costs (undiscounted)	15.5	1.8	6.06	0.144	4.03	1.11	2.39
Health care costs [illegible]	10.5	1.71	5.64	0.111	1.29	0.549	1.23
Societal costs (undiscounted) (I$	11.7	2.55	6.61	0.0996	1.08	0.543	0.81
IPD cases (Millions)	3.12	1.53	1.46	0.00932	0.0285	0.071	0.0186
Non-IPD pneumonia	7.89	3.68	3.91	0.0246	0.0634	0.154	0.0403
Acute otitis media	391	212	143	2.22	12.6	13.6	7.39
Deaths (Millions)	0.772	0.56	0.188	0.000739	0.00495	0.0109	0.00287
DALYs lost	33.4	21.4	10.3	0.0826	0.467	0.705	0.275
**C. DALYs lost (discounted)**	**22.2**	**14**	**6.99**	**0.0552**	**0.319**	**0.483**	**0.185**
Total costs (undiscounted) (I$, Billions)	37.7	6.06	18.3	0.354	6.39	2.2	4.42

**D. Total costs (discounted) (I$, Billions)**	**25.6**	**4**	**12.5**	**0.237**	**4.37**	**1.51**	**2.96**
Total health system costs* (undiscounted) (I$, Billions)	26.1	3.52	11.7	0.254	5.31	1.66	3.61
Total health system costs* (discounted) (I$, Billions)	17.7	2.33	7.99	0.171	3.63	1.14	2.42
**PCV-13 vaccination vs. no PCV vaccination (yearly)**							
**(1) D-B: Incremental cost (discounted) (I$, Billions)**	**6.66**	**0.66**	**2.26**	**0.03**	**2.34**	**0.488**	**0.89**
**(2) A-C: DALYs adverted (discounted) (Millions)**	**9.13**	**5.65**	**2.62**	**0.02**	**0.15**	**0.29**	**0.20**
**ICER overall 30 years**							
**Incremental cost-effectiveness ratio over 30 years ≈ (1)/(2)) due to rounding differences (I$ / DALYs)**	**724.38**	**117.66**	**853.22**	**1588.41**	**16035.54**	**1682.21**	**4424.12**

Table 4b. Global and regional incremental outcomes of PCV-13 compared to no vaccination in one year

	Global	Africa	Asia	Oceania	Europe	LAC	North America
Number of children fully vaccinated (at least 3 doses) (Millions)	114	35	58.1	0.561	8.77	6.7	4.42
Vaccination program costs (undiscounted) (I$, Billions)	15.5	1.8	6.06	0.144	4.03	1.11	2.39
Health care costs (undiscounted) (I$, Billions)	-3.19	-0.364	-1.35	-0.0637	-0.38	-0.222	-0.81
Societal costs (undiscounted) (I$, Billions) [OOP and Productivity]	-2.64	-0.463	-1.46	-0.0297	-0.237	-0.182	-0.262
IPD cases (Millions)	-1.65	-0.757	-0.725	- [illegible]	-0.028	- [illegible]	-0.0487
Non-IPD pneumonia cases (Millions)	-4.14	-1.82	-1.94	-0.0206	-0.0622	-0.151	-0.105
Acute otitis media cases (Millions)	-48.8	-24.5	-19.2	-0.266	-1.85	-1.92	-1.12
Deaths (Millions)	-0.399	-0.275	-	-	-	-	-0.00754
DALYs lost (undiscounted) (Millions)	-13.8	-8.68	-3.88	-0.0328	-0.216	-0.429	-0.301
A. DALYs lost (discounted) (Millions)	-9.13	-5.65	-2.62	-0.0217	-0.146	-0.291	-0.2
Total costs (undiscounted) (I$, Billions)	9.71	0.977	3.25	0.0503	3.41	0.707	1.32
B. Total costs (discounted) (I$, Billions)	6.67	0.663	2.26	0.0345	2.34	0.488	0.886
Total health system costs* (undiscounted) (I$, Billions)	12.3	1.44	4.71	0.08	3.65	0.89	1.58
Total health system costs* (discounted) (I$, Billions)	8.42	0.962	3.24	0.0541	2.5	0.611	1.06
Incremental cost-effectiveness ratio over 30 years ≈ B/-A (I$ / DALYs)	724.4	117.7	853.2	1588.4	16035.5	1682.2	4424.1

IPD: Invasive pneumococcal disease.

*Health system costs include vaccination program costs plus healthcare costs and exclude societal costs (OOP and productivity costs).

Table 5a. Subgroup analyses yearly estimates of pneumococcal disease related outcomes without PCV introduction

	All Gavi AMC eligible	Gavi AMC pending introduction	All countries pending introduction*	High coverage countries scenario	Gavi AMC already introduced
Number of children fully vaccinated (at least 3 doses) (Millions)	67.5	28.1	48.9	54.2	39.4
Vaccination program costs (undiscounted) (I$, Billions)	0	0	0	0	0
Health care costs (undiscounted) (I$, Billions)	5.05	3.02	5.44	7.26	2.03
Societal costs (undiscounted) (I$, Billions) [OOP and Productivity]	8.29	4.92	6.88	5.61	3.37
IPD cases (Millions)	3.70	1.31	1.92	2.02	2.38
Non-IPD pneumonia cases (Millions)	9.33	3.55	5.11	4.89	5.78
Acute otitis media cases (Millions)	352	102	138	202	250
Deaths (Millions)	1	0.183	0.275	0.616	0.822
DALYs lost (undiscounted) (Millions)	39	8.96	13.1	23.5	30.1
DALYs lost (discounted) (Millions)	25.7	6.05	8.87	15.5	19.7
Total costs (undiscounted) (I$, Billions)	13.3	7.94	12.3	12.9	5.41
Total costs (discounted) (I$, Billions)	8.95	5.39	8.38	8.67	3.56
Total health system costs* (undiscounted) (I$, Billions)	5.05	3.02	5.44	7.26	2.03
Total health system costs* (discounted) (I$, Billions)	3.39	2.05	3.71	4.89	1.34
Incremental cost-effectiveness ratio over 30 years (I$ / DALYs)	51.45	-1.874	597.966	1089.725	70.563

*Includes all countries, Gavi AMC and non-Gavi AMC eligible, pending introduction.

Table 5b. Subgroup analyses outcomes of PCV-13 vaccination in one year

	All Gavi AMC eligible	Gavi AMC pending introduction	All countries pending introduction*	High coverage countries scenario	Gavi AMC already introduced
Number of children fully vaccinated (at least 3 doses) (Millions)	67.5	28.1	48.9	54.2	39.4
Vaccination program costs (undiscounted) (I$, Billions)	2.80	1.38	4.42	10.5	1.42
Health care costs (undiscounted) (I$, Billions)	4.17	2.47	4.38	5.29	1.7
Societal costs (undiscounted) (I$, Billions) [OOP and Productivity]	6.9	4.04	5.61	4.49	2.86
IPD cases (Millions)	2.46	0.873	1.27	1.29	1.58
Non-IPD pneumonia cases (Millions)	6.22	2.36	3.4	3.13	3.84
Acute otitis media cases (Millions)	314	90.9	122	178	223
Deaths (Millions)	0.665	0.12	0.181	0.399	0.543
DALYs lost (undiscounted) (Millions)	27.8	6.42	9.32	16.3	21.3
DALYs lost (discounted) (Millions)	18.3	4.35	6.33	10.8	14
Total costs (undiscounted) (I$, Billions)	13.9	7.89	14.4	20.3	5.98
Total costs (discounted) (I$, Billions)	9.33	5.38	9.85	13.7	3.95
Total health system costs* (undiscounted) (I$, Billions)	6.97	3.85	8.8	15.8	3.12
Total health system costs* (discounted) (I$, Billions)	4.69	2.63	6.02	10.7	2.06
Incremental cost-effectiveness ratio over 30 years (I$ / DALYs)	51.45	-1.874	597.966	1089.725	70.563

*Includes all countries, Gavi AMC and non-Gavi AMC eligible, pending introduction.

Table 5c. Incremental outcomes of PCV-13 compared to no vaccination in one year

	All Gavi AMC eligible	Gavi AMC pending introduction	All countries pending introduction*	High coverage countries scenario	Gavi AMC already introduced
Number of children fully vaccinated (at least 3 doses) (Millions)	67.5	28.1	48.9	54.2	39.4
Vaccination program costs (undiscounted) (I$, Billions)	2.8	1.38	4.42	10.5	1.42
Health care costs (undiscounted) (I$, Billions)	-0.88	-0.543	-1.05	-1.98	--0.333
Societal costs (undiscounted) (I$, Billions) [OOP and Productivity]	-1.39	-0.869	-1.26	-1.13	-0.511
IPD cases (Millions)	-1.23	-0.435	-0.638	-0.723	-0.789
Non-IPD pneumonia cases (Millions)	-3.12	-1.17	-1.7	-1.74	-1.92
Acute otitis media cases (Millions)	-37.8	-11.6	-16.1	-24.4	-26.2
Deaths (Millions)	-0.33	-0.0597	-0.09	-0.208	-0.267
DALYs lost (undiscounted) (Millions)	-11.1	-2.43	-3.65	-7.07	-8.52
DALYs lost (discounted) (Millions)	-7.29	-1.63	-2.46	-4.65	-5.56
Total costs (undiscounted) (I$, Billions)	0.534	-0.0332	2.11	7.42	0.576
Total costs (discounted) (I$, Billions)	0.386	-0.00285	1.48	5.06	0.392
Total health system costs* (undiscounted) (I$, Billions)	1.92	0.836	3.37	8.55	1.09
Total health system costs* (discounted) (I$, Billions)	1.3	0.578	2.32	5.81	0.725
Incremental cost-effectiveness ratio over 30 years (I$ / DALYs)	51.45	-1.874	597.966	1089.725	70.563

*Includes all countries, Gavi AMC and non-Gavi AMC eligible, pending introduction.

Table 5d. Estimated number of death preventable in the five countries with largest populations of children under 5 years old, that are pending vaccine introduction.

Country	Number of deaths prevented ('000)	Death prevented per 100,000 (accounts for the U5 population)
India	1090	135
China	265	56.5
Indonesia	211	134
Egypt	168	205
Algeria	150	562

Table 5e. DALYs averted and ICER for introducing PCV13 compared to no vaccination for each region, with only direct effects of vaccination and with indirect effects (herd protection and serotype replacement).

Region	DALYs averted (discounted)			ICER		
	Direct effects only	With indirect effects	Difference	Direct effects only	With indirect effects	Difference
Asia	111,000,000	78,600,000	32,800,000	$446	$853	-$407
Africa	230,000,000	170,000,000	60,700,000	$73	$118	-$45
Oceania	668,000	650,000	17,800	$1,150	$1,590	-$440
Latin America	8,740,000	8,730,000	8,470	$1,650	$1,680	-$30
Europe	4,570,000	4,370,000	202,000	$15,200	$16,000	-$800
North America	4,510,000	6,000,000	-1,490,000	$6,730	$4,420	$2,310

(in 3 significant figures)

3.4. Budget Impact Analysis

As per our budget impact analysis, Table 6 presents the yearly global undiscounted costs of vaccination program, healthcare (including hospital admissions and heath centre visits), health systems (healthcare and vaccination program) and total costs of PCV-13 vaccination. Table 7a, 7b, 7c, 7d, 7e, and 7f present the yearly UN regional estimates of these outcomes. All results are in billion international dollars (I$ Billion).

Table 6. Global costs of PCV-13 vaccination

Year	Total vaccination program costs			Total healthcare costs			Total health system costs		
	NO PCV	PCV 13	PCV Diff	NO PCV	PCV 13	PCV Diff	NO PCV	PCV 13	PCV Diff
Total	0	466	466	412	316	-95.7	412	782	370
1	0	19	19	14.1	12.1	-1.95	14.1	31.2	17.1
2	0	16.2	16.2	14.1	11.4	-2.69	14.1	27.6	13.5
3	0	16.2	16.2	14.1	11.1	-2.94	14.1	27.3	13.2
4	0	16.2	16.2	14.1	10.9	-3.17	14.1	27	13
5	0	16.1	16.1	14.1	10.7	-3.36	14.1	26.8	12.8
6	0	16	16	14	10.6	-3.35	14	26.7	12.7
7	0	15.9	15.9	13.9	10.6	-3.34	13.9	26.5	12.6
8	0	15.8	15.8	13.9	10.6	-3.32	13.9	26.4	12.5
9	0	15.7	15.7	13.8	10.5	-3.31	13.8	26.2	12.4
10	0	15.6	15.6	13.8	10.5	-3.3	13.8	26.1	12.3
11	0	15.5	15.5	13.7	10.4	-3.29	13.7	25.9	12.2
12	0	15.4	15.4	13.7	10.4	-3.28	13.7	25.8	12.1
13	0	15.3	15.3	13.6	10.4	-3.27	13.6	25.7	12
14	0	15.2	15.2	13.6	10.3	-3.26	13.6	25.6	12
15	0	15.1	15.1	13.6	10.3	-3.25	13.6	25.4	11.9
16	0	15.1	15.1	13.6	10.3	-3.24	13.6	25.4	11.9
17	0	15.1	15.1	13.5	10.3	-3.24	13.5	25.4	11.8
18	0	15	15	13.5	10.3	-3.24	13.5	25.3	11.8
19	0	15	15	13.5	10.3	-3.23	13.5	25.3	11.8
20	0	15	15	13.5	10.3	-3.23	13.5	25.3	11.8
21	0	15	15	13.5	10.3	-3.23	13.5	25.3	11.8
22	0	15	15	13.5	10.3	-3.24	13.5	25.3	11.8
23	0	15.1	15.1	13.6	10.3	-3.24	13.6	25.4	11.8
24	0	15.1	15.1	13.6	10.3	-3.24	13.6	25.4	11.9
25	0	15.1	15.1	13.6	10.3	-3.25	13.6	25.5	11.9
26	0	15.2	15.2	13.6	10.3	-3.25	13.6	25.5	11.9
27	0	15.2	15.2	13.6	10.3	-3.26	13.6	25.5	11.9
28	0	15.2	15.2	13.6	10.4	-3.26	13.6	25.6	12
29	0	15.3	15.3	13.6	10.4	-3.26	13.6	25.6	12
30	0	15.3	15.3	13.6	10.4	-3.26	13.6	25.7	12

Table 7a. Costs of PCV-13 vaccination in Asia

Year	Total vaccination program costs			Total healthcare costs			Total health system costs		
	NO PCV	PCV 13	Difference	NO PCV	PCV 13	Difference	NO PCV	PCV 13	Difference
Total	0	182	182	210	169	-40.5	210	351	141
1	0	7.74	7.74	7.62	6.79	-0.823	7.62	14.5	6.91
2	0	6.58	6.58	7.59	6.42	-1.16	7.59	13	5.42
3	0	6.54	6.54	7.55	6.26	-1.29	7.55	12.8	5.26
4	0	6.51	6.51	7.52	6.11	-1.4	7.52	12.6	5.1
5	0	6.47	6.47	7.49	5.98	-1.5	7.49	12.4	4.97
6	0	6.4	6.4	7.42	5.92	-1.49	7.42	12.3	4.92
7	0	6.33	6.33	7.35	5.87	-1.47	7.35	12.2	4.86
8	0	6.27	6.27	7.28	5.82	-1.46	7.28	12.1	4.81
9	0	6.2	6.2	7.21	5.76	-1.44	7.21	12	4.76
10	0	6.13	6.13	7.14	5.71	-1.43	7.14	11.8	4.7
11	0	6.08	6.08	7.09	5.67	-1.42	7.09	11.8	4.67
12	0	6.04	6.04	7.04	5.63	-1.4	7.04	11.7	4.63
13	0	5.99	5.99	6.99	5.59	-1.39	6.99	11.6	4.6
14	0	5.95	5.95	6.94	5.55	-1.38	6.94	11.5	4.56
15	0	5.9	5.9	6.89	5.51	-1.37	6.89	11.4	4.53
16	0	5.88	5.88	6.87	5.49	-1.37	6.87	11.4	4.51
17	0	5.86	5.86	6.84	5.47	-1.36	6.84	11.3	4.5
18	0	5.84	5.84	6.82	5.45	-1.36	6.82	11.3	4.49
19	0	5.82	5.82	6.79	5.43	-1.35	6.79	11.3	4.47
20	0	5.81	5.81	6.77	5.41	-1.35	6.77	11.2	4.46
21	0	5.8	5.8	6.75	5.4	-1.34	6.75	11.2	4.45
22	0	5.79	5.79	6.73	5.39	-1.34	6.73	11.2	4.45
23	0	5.78	5.78	6.72	5.37	-1.34	6.72	11.1	4.44
24	0	5.77	5.77	6.7	5.36	-1.33	6.7	11.1	4.44
25	0	5.76	5.76	6.68	5.35	-1.33	6.68	11.1	4.43
26	0	5.75	5.75	6.67	5.33	-1.32	6.67	11.1	4.42
27	0	5.73	5.73	6.65	5.32	-1.32	6.65	11.1	4.41
28	0	5.72	5.72	6.63	5.31	-1.32	6.63	11	4.41
29	0	5.71	5.71	6.61	5.29	-1.31	6.61	11	4.4
30	0	5.7	5.7	6.6	5.28	-1.31	6.6	11	4.39

Table 7b. Costs of PCV-13 vaccination in Africa

Year	Total vaccination program costs			Total healthcare costs			Total health system costs		
	NO PCV	PCV 13	Difference	NO PCV	PCV 13	Difference	NO PCV	PCV 13	Difference
Total	0	54.1	54.1	62.3	51.4	-10.9	62.3	105	43.2
1	0	2.06	2.06	1.79	1.61	-0.179	1.79	3.67	1.89
2	0	1.63	1.63	1.81	1.55	-0.255	1.81	3.18	1.38
3	0	1.64	1.64	1.82	1.54	-0.286	1.82	3.18	1.36
4	0	1.65	1.65	1.84	1.53	-0.314	1.84	3.18	1.34
5	0	1.66	1.66	1.86	1.52	-0.34	1.86	3.18	1.32
6	0	1.67	1.67	1.88	1.54	-0.343	1.88	3.2	1.33
7	0	1.68	1.68	1.9	1.55	-0.345	1.9	3.22	1.33
8	0	1.68	1.68	1.91	1.56	-0.347	1.91	3.25	1.34
9	0	1.69	1.69	1.93	1.58	-0.35	1.93	3.27	1.34
10	0	1.7	1.7	1.95	1.59	-0.352	1.95	3.29	1.35
11	0	1.71	1.71	1.97	1.61	-0.355	1.97	3.32	1.35
12	0	1.72	1.72	1.99	1.63	-0.358	1.99	3.35	1.36
13	0	1.73	1.73	2.01	1.64	-0.361	2.01	3.37	1.37
14	0	1.74	1.74	2.03	1.66	-0.365	2.03	3.4	1.38
15	0	1.75	1.75	2.05	1.68	-0.368	2.05	3.43	1.38
16	0	1.77	1.77	2.07	1.7	-0.371	2.07	3.47	1.4
17	0	1.79	1.79	2.1	1.72	-0.375	2.1	3.51	1.41
18	0	1.8	1.8	2.12	1.74	-0.379	2.12	3.54	1.42
19	0	1.82	1.82	2.14	1.76	-0.383	2.14	3.58	1.44
20	0	1.84	1.84	2.17	1.78	-0.387	2.17	3.62	1.45
21	0	1.86	1.86	2.19	1.8	-0.391	2.19	3.66	1.47
22	0	1.88	1.88	2.22	1.82	-0.396	2.22	3.7	1.48
23	0	1.9	1.9	2.24	1.84	-0.4	2.24	3.74	1.5
24	0	1.92	1.92	2.27	1.86	-0.404	2.27	3.78	1.51
25	0	1.93	1.93	2.29	1.88	-0.408	2.29	3.82	1.53
26	0	1.95	1.95	2.31	1.9	-0.412	2.31	3.85	1.54
27	0	1.96	1.96	2.33	1.92	-0.415	2.33	3.88	1.55
28	0	1.98	1.98	2.35	1.93	-0.419	2.35	3.91	1.56
29	0	1.99	1.99	2.38	1.95	-0.422	2.38	3.95	1.57
30	0	2.01	2.01	2.4	1.97	-0.426	2.4	3.98	1.58

Table 7c. Costs of PCV-13 vaccination in Oceania

Year	Total vaccination program costs			Total healthcare costs			Total health system costs		
	NO PCV	PCV 13	Difference	NO PCV	PCV 13	Difference	NO PCV	PCV 13	Difference
Total	0	4.31	4.31	5.23	3.32	-1.91	5.23	7.63	2.4
1	0	0.16	0.16	0.162	0.119	-0.0438	0.162	0.278	0.116
2	0	0.136	0.136	0.165	0.107	-0.0575	0.165	0.243	0.0786
3	0	0.138	0.138	0.167	0.107	-0.0596	0.167	0.245	0.0781
4	0	0.139	0.139	0.169	0.108	-0.0617	0.169	0.247	0.0777
5	0	0.141	0.141	0.172	0.108	-0.0636	0.172	0.249	0.0775
6	0	0.141	0.141	0.172	0.108	-0.0637	0.172	0.25	0.0776
7	0	0.141	0.141	0.172	0.109	-0.0638	0.172	0.25	0.0777
8	0	0.142	0.142	0.173	0.109	-0.0638	0.173	0.25	0.0778
9	0	0.142	0.142	0.173	0.109	-0.0639	0.173	0.251	0.0779
10	0	0.142	0.142	0.173	0.109	-0.064	0.173	0.251	0.0779
11	0	0.142	0.142	0.173	0.109	-0.064	0.173	0.251	0.0779
12	0	0.142	0.142	0.173	0.109	-0.0639	0.173	0.251	0.0779
13	0	0.142	0.142	0.173	0.109	-0.0639	0.173	0.251	0.0779
14	0	0.142	0.142	0.173	0.109	-0.0639	0.173	0.251	0.0779
15	0	0.142	0.142	0.173	0.109	-0.0639	0.173	0.251	0.0779
16	0	0.142	0.142	0.173	0.109	-0.064	0.173	0.251	0.078
17	0	0.142	0.142	0.173	0.109	-0.064	0.173	0.251	0.078
18	0	0.142	0.142	0.173	0.109	-0.0641	0.173	0.251	0.0781
19	0	0.142	0.142	0.174	0.109	-0.0642	0.174	0.252	0.0781
20	0	0.142	0.142	0.174	0.11	-0.0642	0.174	0.252	0.0782
21	0	0.143	0.143	0.175	0.11	-0.0646	0.175	0.253	0.0785
22	0	0.144	0.144	0.176	0.111	-0.065	0.176	0.255	0.0789
23	0	0.145	0.145	0.177	0.111	-0.0653	0.177	0.256	0.0792
24	0	0.145	0.145	0.178	0.112	-0.0657	0.178	0.257	0.0795
25	0	0.146	0.146	0.179	0.113	-0.0661	0.179	0.259	0.0799
26	0	0.147	0.147	0.18	0.114	-0.0667	0.18	0.261	0.0804
27	0	0.148	0.148	0.182	0.115	-0.0672	0.182	0.263	0.081
28	0	0.149	0.149	0.183	0.116	-0.0678	0.183	0.265	0.0815
29	0	0.151	0.151	0.185	0.116	-0.0684	0.185	0.267	0.0821
30	0	0.152	0.152	0.187	0.117	-0.069	0.187	0.269	0.0827

Table 7d. Costs of PCV-13 vaccination in Latin America

Year	Total vaccination program costs			Total healthcare costs			Total health system costs		
	NO PCV	PCV 13	Difference	NO PCV	PCV 13	Difference	NO PCV	PCV 13	Difference
Total	0	33.4	33.4	23.1	16.5	-6.67	23.1	49.8	26.7
1	0	1.4	1.4	0.839	0.68	-0.159	0.839	2.08	1.25
2	0	1.2	1.2	0.836	0.624	-0.212	0.836	1.82	0.986
3	0	1.19	1.19	0.832	0.608	-0.224	0.832	1.8	0.969
4	0	1.19	1.19	0.829	0.595	-0.234	0.829	1.78	0.953
5	0	1.18	1.18	0.825	0.582	-0.243	0.825	1.76	0.939
6	0	1.18	1.18	0.82	0.579	-0.242	0.82	1.75	0.934
7	0	1.17	1.17	0.816	0.575	-0.24	0.816	1.74	0.929
8	0	1.16	1.16	0.811	0.572	-0.239	0.811	1.73	0.923
9	0	1.16	1.16	0.806	0.569	-0.238	0.806	1.72	0.918
10	0	1.15	1.15	0.801	0.565	-0.236	0.801	1.71	0.913
11	0	1.14	1.14	0.796	0.561	-0.235	0.796	1.7	0.906
12	0	1.13	1.13	0.79	0.557	-0.233	0.79	1.69	0.9
13	0	1.13	1.13	0.785	0.553	-0.232	0.785	1.68	0.893
14	0	1.12	1.12	0.779	0.549	-0.23	0.779	1.67	0.887
15	0	1.11	1.11	0.774	0.546	-0.228	0.774	1.65	0.881
16	0	1.1	1.1	0.768	0.542	-0.227	0.768	1.64	0.875
17	0	1.09	1.09	0.763	0.538	-0.225	0.763	1.63	0.868
18	0	1.09	1.09	0.758	0.534	-0.224	0.758	1.62	0.862
19	0	1.08	1.08	0.752	0.53	-0.222	0.752	1.61	0.856
20	0	1.07	1.07	0.747	0.526	-0.22	0.747	1.6	0.85
21	0	1.06	1.06	0.742	0.523	-0.219	0.742	1.59	0.844
22	0	1.06	1.06	0.737	0.519	-0.218	0.737	1.58	0.839
23	0	1.05	1.05	0.732	0.516	-0.216	0.732	1.57	0.833
24	0	1.04	1.04	0.727	0.513	-0.215	0.727	1.55	0.828
25	0	1.04	1.04	0.722	0.509	-0.213	0.722	1.54	0.822
26	0	1.03	1.03	0.718	0.506	-0.212	0.718	1.54	0.817
27	0	1.02	1.02	0.714	0.503	-0.211	0.714	1.53	0.812
28	0	1.02	1.02	0.709	0.5	-0.21	0.709	1.52	0.808
29	0	1.01	1.01	0.705	0.497	-0.208	0.705	1.51	0.803
30	0	1.01	1.01	0.701	0.494	-0.207	0.701	1.5	0.798

Table 7e. Costs of PCV-13 vaccination in Europe

Year	Total vaccination program costs			Total healthcare costs			Total health system costs		
	NO PCV	PCV 13	Difference	NO PCV	PCV 13	Difference	NO PCV	PCV 13	Difference
Total	0	121	121	50	38.7	-11.4	50	159	109
1	0	5.53	5.53	1.76	1.54	-0.222	1.76	7.07	5.31
2	0	4.42	4.42	1.75	1.44	-0.313	1.75	5.87	4.11
3	0	4.4	4.4	1.75	1.4	-0.351	1.75	5.8	4.04
4	0	4.37	4.37	1.75	1.37	-0.383	1.75	5.73	3.98
5	0	4.34	4.34	1.74	1.33	-0.411	1.74	5.67	3.93
6	0	4.29	4.29	1.73	1.32	-0.409	1.73	5.61	3.88
7	0	4.24	4.24	1.72	1.32	-0.406	1.72	5.55	3.83
8	0	4.19	4.19	1.71	1.31	-0.404	1.71	5.5	3.78
9	0	4.14	4.14	1.7	1.3	-0.401	1.7	5.44	3.73
10	0	4.08	4.08	1.69	1.29	-0.399	1.69	5.38	3.69
11	0	4.03	4.03	1.68	1.28	-0.396	1.68	5.32	3.64
12	0	3.98	3.98	1.67	1.28	-0.393	1.67	5.26	3.59
13	0	3.93	3.93	1.66	1.27	-0.39	1.66	5.2	3.54
14	0	3.88	3.88	1.64	1.26	-0.387	1.64	5.14	3.49
15	0	3.82	3.82	1.63	1.25	-0.385	1.63	5.07	3.44
16	0	3.8	3.8	1.63	1.25	-0.383	1.63	5.05	3.42
17	0	3.78	3.78	1.62	1.24	-0.382	1.62	5.02	3.4
18	0	3.76	3.76	1.62	1.24	-0.381	1.62	5	3.38
19	0	3.74	3.74	1.61	1.23	-0.38	1.61	4.98	3.36
20	0	3.72	3.72	1.61	1.23	-0.379	1.61	4.95	3.34
21	0	3.74	3.74	1.61	1.23	-0.38	1.61	4.97	3.36
22	0	3.76	3.76	1.62	1.24	-0.38	1.62	4.99	3.38
23	0	3.78	3.78	1.62	1.24	-0.381	1.62	5.02	3.39
24	0	3.79	3.79	1.62	1.24	-0.382	1.62	5.04	3.41
25	0	3.81	3.81	1.63	1.25	-0.383	1.63	5.06	3.43
26	0	3.84	3.84	1.63	1.25	-0.385	1.63	5.09	3.45
27	0	3.87	3.87	1.64	1.25	-0.386	1.64	5.12	3.48
28	0	3.89	3.89	1.64	1.26	-0.388	1.64	5.15	3.51
29	0	3.92	3.92	1.65	1.26	-0.389	1.65	5.18	3.53
30	0	3.95	3.95	1.66	1.27	-0.39	1.66	5.21	3.56

Table 7f. Costs of PCV-13 vaccination in North America

Year	Total vaccination program costs			Total healthcare costs			Total health system costs		
	NO PCV	PCV 13	Difference	NO PCV	PCV 13	Difference	NO PCV	PCV 13	Difference
Total	0	71.7	71.7	61.1	36.8	-24.3	61.1	108	47.3
1	0	2.15	2.15	1.9	1.38	-0.524	1.9	3.53	1.62
2	0	2.26	2.26	1.92	1.23	-0.697	1.92	3.49	1.56
3	0	2.28	2.28	1.94	1.21	-0.734	1.94	3.49	1.55
4	0	2.3	2.3	1.96	1.19	-0.769	1.96	3.49	1.53
5	0	2.32	2.32	1.98	1.18	-0.8	1.98	3.5	1.52
6	0	2.34	2.34	1.99	1.18	-0.805	1.99	3.52	1.53
7	0	2.35	2.35	2	1.19	-0.809	2	3.54	1.54
8	0	2.36	2.36	2.01	1.2	-0.813	2.01	3.56	1.55
9	0	2.37	2.37	2.02	1.2	-0.818	2.02	3.58	1.56
10	0	2.39	2.39	2.03	1.21	-0.822	2.03	3.6	1.56
11	0	2.39	2.39	2.04	1.21	-0.823	2.04	3.6	1.57
12	0	2.39	2.39	2.04	1.21	-0.825	2.04	3.61	1.57
13	0	2.4	2.4	2.04	1.21	-0.826	2.04	3.61	1.57
14	0	2.4	2.4	2.04	1.22	-0.827	2.04	3.62	1.57
15	0	2.4	2.4	2.05	1.22	-0.829	2.05	3.62	1.58
16	0	2.41	2.41	2.05	1.22	-0.829	2.05	3.63	1.58
17	0	2.41	2.41	2.05	1.22	-0.83	2.05	3.63	1.58
18	0	2.41	2.41	2.05	1.22	-0.831	2.05	3.63	1.58
19	0	2.41	2.41	2.05	1.22	-0.831	2.05	3.63	1.58
20	0	2.41	2.41	2.06	1.22	-0.832	2.06	3.64	1.58
21	0	2.42	2.42	2.06	1.23	-0.834	2.06	3.65	1.59
22	0	2.43	2.43	2.07	1.23	-0.836	2.07	3.66	1.59
23	0	2.43	2.43	2.07	1.23	-0.838	2.07	3.66	1.59
24	0	2.44	2.44	2.08	1.24	-0.84	2.08	3.67	1.6
25	0	2.44	2.44	2.08	1.24	-0.842	2.08	3.68	1.6
26	0	2.45	2.45	2.09	1.24	-0.845	2.09	3.69	1.61
27	0	2.46	2.46	2.09	1.25	-0.847	2.09	3.71	1.61
28	0	2.47	2.47	2.1	1.25	-0.85	2.1	3.72	1.62
29	0	2.48	2.48	2.11	1.25	-0.853	2.11	3.73	1.62
30	0	2.48	2.48	2.11	1.26	-0.855	2.11	3.74	1.63

4. REFERENCES

1 Drummond MF, Sculpher MJ, Torrance GW, O'Brien BJ, Stoddart GL. Methods for the Economic Evaluation of healthcare Programmes, Third Edit. Oxford: Oxford University Press, 2005.

2 Flasche S, Le Polain de Waroux O, O'Brien KL, Edmunds WJ. The serotype distribution among healthy carriers before vaccination is essential for predicting the impact of pneumococcal conjugate vaccine on invasive disease. *PLoS Comput Biol* 2015; **11**: 1–15.

3 Feikin DR, Kagucia EW, Loo JD, *et al.* Serotype-Specific Changes in Invasive Pneumococcal Disease after Pneumococcal Conjugate Vaccine Introduction: A Pooled Analysis of Multiple Surveillance Sites. *PLoS Med* 2013; **10**: e1001517.

4 Weinbergera M D, Malley R, Lipsitch M. Serotype replacement in disease following pneumococcal vaccination: A discussion of the evidence. *Lancet* 2012; **378**: 1962–73.

5 Hammitt LL, Akech DO, Morpeth SC, *et al.* Population effect of 10-valent pneumococcal conjugate vaccine on nasopharyngeal carriage of Streptococcus pneumoniae and non-typeable Haemophilus influenzae in Kilifi, Kenya: Findings from cross-sectional carriage studies. *Lancet Glob Heal* 2014; **2**: e397–405.

6 Waight PA, Andrews NJ, Ladhani SN, Sheppard CL, Slack MPE, Miller E. Effect of the 13-valent pneumococcal conjugate vaccine on invasive pneumococcal disease in England and Wales 4 years after its introduction: an observational cohort study. *Lancet Infect Dis* 2015; **15**: 535–43.

7 Bruce MG, Singleton R, Bulkow L, *et al.* Impact of the 13-valent pneumococcal conjugate vaccine (pcv13) on invasive pneumococcal disease and carriage in Alaska. *Vaccine* 2015; **33**: 4813–9.

8 Flasche S, Givon-Lavi N, Dagan R. Using Pneumococcal Carriage Data to Monitor Postvaccination Changes in the Incidence of Pneumococcal Otitis Media. *Am J Epidemiol* 2016. DOI:10.1093/aje/kww012.

9 Tregnaghi MW, Sáez-Llorens X, López P, *et al.* Efficacy of pneumococcal nontypable Haemophilus influenzae protein D conjugate vaccine (PHiD-CV) in young Latin American children: A double-blind randomized controlled trial. *PLoS Med* 2014; **11**: e1001657.

10 Johnson HL, Deloria-Knoll M, Levine OS, *et al.* Systematic evaluation of serotypes causing invasive pneumococcal disease among children under five: the pneumococcal global serotype project. *PLoS Med* 2010; **7**. DOI:10.1371/journal.pmed.1000348.

11 O. le Polain de Waroux and S. Flasche, personal communication. .

12 World Bank. GDP per capita, PPP (current international $). World Bank Data Bank. 2016. http://data.worldbank.org/indicator/NY.GDP.PCAP.PP.CD (accessed Dec 1, 2016).

13 International Monetary Fund. Gross domestic product based on purchasing-power-parity (PPP) per capita GDP. World Econ. Outlook Database. 2016. https://www.imf.org/external/pubs/ft/weo/2016/02/weodata/index.aspx (accessed Dec 1, 2016).

14 Gavi. Advanced Market Commitment for pneumococcal vaccines - Annual Report. Geneva, 2015.

15 World Health Organization. Immunization schedules. WHO vaccine-preventable Dis. Monit. Syst. Glob. Summ. 2016. http://apps.who.int/immunization_monitoring/globalsummary/schedules (accessed Dec 8, 2016).

16 United Nations Population Division. World populations prospects, the 2015 Revision -Total Population - Both Sexes. World Popul. Prospect. 2015. 2015. https://esa.un.org/unpd/wpp/Download/Standard/Population/ (accessed Jan 1, 2016).

17 United Nations Population Division. World Populations prospects, the 2015 Revision - Deaths By age groups. World Popul. Prospect. 2015. https://esa.un.org/unpd/wpp/Download/Standard/Mortality/ (accessed Nov 11, 2016).
18 O'Brien KL, Wolfson LJ, Watt JP, *et al.* Burden of disease caused by Streptococcus pneumoniae in children younger than 5 years: global estimates. *Lancet* 2009; **374**: 893–902.
19 Said MA, Johnson HL, Nonyane BAS, Deloria-Knoll M, OBrien KL. Estimating the Burden of Pneumococcal Pneumonia among Adults: A Systematic Review and Meta-Analysis of Diagnostic Techniques. *PLoS One* 2013; **8**. DOI:10.1371/journal.pone.0060273.
20 Cutts FT, Zaman SMA, Enwere G ym, *et al.* Efficacy of nine-valent pneumococcal conjugate vaccine against pneumonia and invasive pneumococcal disease in The Gambia: randomised, double-blind, placebo-controlled trial. *Lancet* 2005; **365**: 1139–46.
21 Monasta L, Ronfani L, Marchetti F, *et al.* Burden of disease caused by otitis media: systematic review and global estimates. *PLoS One* 2012; **7**: e36226.
22 Mathers CD, Lopez AD, Murray CJL. Chapter 3: The Burden of Disease and Mortality by Condition : Data , Methods , and Results for 2001. *Glob Burd Dis Risk Factors* 2006; **2003**: 45–93.
23 Vos T, Allen C, Arora M, *et al.* Global, regional, and national incidence, prevalence, and years lived with disability for 310 diseases and injuries, 1990-2015: a systematic analysis for the Global Burden of Disease Study 2015. *Lancet* 2016; **388**: 1545–602.
24 Edmond K, Clark A, Korczak VS, Sanderson C, Griffiths UK, Rudan I. Global and regional risk of disabling sequelae from bacterial meningitis: a systematic review and meta-analysis. *Lancet Infect Dis* 2010; **10**: 317–28.
25 World Health Organization. -Pneumococcal vaccines WHO position paper – 2012 In. *Wkly Epidemiol Rec Relev épidémiologique Hebd* 2012; **88**: 129–44.
26 Whitney CG, Goldblatt D. Dosing Schedules for Pneumococcal Conjugate Vaccine Considerations for Policy Makers. *Pediatr Infect Dis J* 2014; **33**: 172–81.
27 World Health Organization. WHO-UNICEF estimates of DTP3 coverage. WHO vaccine-preventable Dis. Monit. Syst. 2016 Glob. Summ. 2016. http://apps.who.int/immunization_monitoring/globalsummary/timeseries/tswucoveragedtp3.html (accessed Dec 1, 2016).
28 Centers for Disease Control and Prevention - CDC. Vaccination Coverage Among Children Aged 19–35 Months — United States, 2015. Morb. Mortal. Wkly. Rep. 2016; : Weekly / October 7, 2016 / 65(39);1065–1071.
29 World Bank. World Bank Country and Lending Groups. Data. 2016. https://datahelpdesk.worldbank.org/knowledgebase/articles/906519-world-bank-country-and-lending-groups (accessed Dec 1, 2016).
30 Centers for Disease Control and Prevention - CDC. Pediatric/VFC Vaccine Price List. Vaccines Child. Progr. 2016. https://www.cdc.gov/vaccines/programs/vfc/awardees/vaccine-management/price-list/ (accessed Dec 1, 2016).
31 Pan American Health Organization. Expanded Program of Immunization vaccine prices for year 2015 - Amendment I. PAHO Revolv. Fund. 2014. http://www.paho.org/hq/index.php?option=com_content&view=article&id=1864&Itemid=40713&lang=en (accessed Dec 1, 2016).
32 Gavi. Gavi welcomes new record low price for pneumococcal vaccine. Statements. 2016. http://www.gavi.org/library/news/statements/2016/gavi-welcomes-new-record-low-price-for-pneumococcal-vaccine/ (accessed Dec 1, 2015).
33 Gavi. Gavi Advanced Market Commitment. 2014. http://www.gavi.org/funding/pneumococcal-amc/how-the-pneumococcal-amc-works/.

34 World Health Organization. Vaccine Introduction Guidelines. 2005.
35 Parmar D, Baruwa EM, Zuber P, Kone S. Impact of wastage on single and multi-dose vaccine vials: Implications for introducing pneumococcal vaccines in developing countries. *Hum Vaccin* 2010; **6**: 270–8.
36 Gavi. Vaccine Introduction Grant and Operational Support for Campaigns Policy. Geneva, 2012.
37 World Health Organization. Guidelines for estimating costs of introducing new vaccines into the national immunization system. 2002.
38 UNICEF Supply Division. Cold Chain Weight and Volume Calculator. 2015. http://www.unicef.org/supply/index_51098.html.
39 Usuf E, Mackenzie G, Lowe-Jallow Y, *et al.* Costs of vaccine delivery in the Gambia before and after, pentavalent and pneumococcal conjugate vaccine introductions. *Vaccine* 2014; **32**: 1975–81.
40 Griffiths UK, Clark A, Hajjeh R. Cost-effectiveness of Haemophilus influenzae type b conjugate vaccine in low- and middle-income countries: regional analysis and assessment of major determinants. *J Pediatr* 2013; **163**: S50–S59.e9.
41 UNICEF Supply Division. Auto-Disable Syringes, Re-Use Prevention Syringes and Safety Boxes price data. Supplies Logist. 2016. https://www.unicef.org/supply/files/Auto-Disable_(AD)_and_Re-Use_Prevention_(RUP)_Syringes_and_Safety_Boxes_price_data_-_April_2016.pdf (accessed Dec 1, 2016).
42 International Labour Organization. Wages and hours of work in 159 Occupations. LABORSTA - ILOSTAT. 2010. http://laborsta.ilo.org/STP/guest.
43 World Health Organization. Global Health Expenditure database 1995 - 2010. Knoema - WHO. 2012. https://knoema.com/WHOGHEDB2012/global-health-expenditure-database-1995-2010 (accessed Aug 10, 2016).
44 Tijdens K, Vries DH De, Steinmetz S. Health workforce remuneration : comparing wage levels , ranking , and dispersion of 16 occupational groups in 20 countries. 2013; : 1–15.
45 UNICEF. Pneumonia symptoms - Care seeking: Children under 5 with symptoms of pneumonia taken to a health care provider - Percentage. Glob. Databases. 2016. https://data.unicef.org/topic/child-health/pneumonia/ (accessed Dec 1, 2016).
46 UNICEF. Care seeking for fever: Children under 5 with fever in the last two weeks for whom advice or treatment was sought - Percentage. Glob. Databases. 2016. https://data.unicef.org/wp-content/uploads/2015/12/child-health_malaria_care-seeking-for-fever_feb-2016-1.xlsx (accessed Dec 1, 2016).
47 Wolleswinkel-van den Bosch JH, Stolk EA, Francois M, Gasparini R, Brosa M. The health care burden and societal impact of acute otitis media in seven European countries: Results of an Internet survey. *Vaccine* 2010; **28**, **Supple**: G39–52.
48 World Health Organization. WHO-CHOICE unit cost estimates for service delivery. Cost Eff. Strateg. Plan. 2011. http://www.who.int/choice/cost-effectiveness/inputs/health_service/en/.
49 Mongelluzzo J, Mohamad Z, Ten Have TR SS. Impact of bacterial meningitis-associated conditions on pediatric inpatient resource utilization. *J Hosp Med* 2010; **5**: E1-7.
50 Witt WP, Weiss AJ, Elixhauser A. Overview of Hospital Stays for Children in the United States, 2012. Stat. Br. #187. 2014. https://www.hcup-us.ahrq.gov/reports/statbriefs/sb187-Hospital-Stays-Children-2012.jsp (accessed Dec 1, 2016).
51 Owens PL, Thompson J, Elixhauser A, Ryan K. Care of Children and Adolescents in U.S. Hospitals. Rockville, MD. USA, 2003.
52 Levit K, Stranges E, Ryan K, Elixhauser A. Inpatient Hospital Stays for Principal

Diagnosis*: Average Length of Stay and Average Charges, 2006. Rockville, MD. USA, 2008.
53 Saokaew S, Rayanakorn A, Wu DB-C, Chaiyakunapruk N. Cost Effectiveness of Pneumococcal Vaccination in Children in Low- and Middle-Income Countries: A Systematic Review. *Pharmacoeconomics* 2016; **34**: 1211–25.
54 Giglio N, Micone P, Gentile A. The pharmacoeconomics of pneumococcal conjugate vaccines in Latin America. *Vaccine* 2011; **29**: C35–42.
55 Giachetto Larraz G, Telechea Ortiz H, Speranza Mourine N, *et al.* Costo-efectividad de la vacunación universal antineumocócica en Uruguay. *Rev Panam Salud Pública* 2010; **28**: 92–9.
56 Haasis MA, Ceria JA, Kulpeng W, Teerawattananon Y, Alejandria M. Do pneumococcal conjugate vaccines represent good value for money in a lower-middle income country? A cost-utility analysis in the Philippines. *PLoS One* 2015; **10**: 1–20.
57 Hoshi S ling, Kondo M, Okubo I. Economic evaluation of vaccination programme of 13-valent pneumococcal conjugate vaccine to the birth cohort in Japan. *Vaccine* 2013; **31**: 2762–71.
58 Kim SY, Lee G, Goldie SJ. Economic evaluation of pneumococcal conjugate vaccination in The Gambia. *BMC Infect Dis* 2010; **10**: 260.
59 Klok RM, Lindkvist RM, Ekelund M, Farkouh RA, Strutton DR. Cost-Effectiveness of a 10- Versus 13-Valent Pneumococcal Conjugate Vaccine in Denmark and Sweden. *Clin Ther* 2013; **35**: 119–34.
60 Komakhidze T, Hoestlandt C, Dolakidze T, *et al.* Cost-effectiveness of pneumococcal conjugate vaccination in Georgia. *Vaccine* 2015; **33**: A219–26.
61 Maurer KA, Chen HF, Wagner AL, *et al.* Cost-effectiveness analysis of pneumococcal vaccination for infants in China. *Vaccine* 2016; **34**: 6343–9.
62 Mezones-Holguin E, Canelo-Aybar C, Clark AD, *et al.* Cost-effectiveness analysis of 10- and 13-valent pneumococcal conjugate vaccines in Peru. *Vaccine* 2015; **33**: A154–66.
63 Newall AT, Creighton P, Philp DJ, Wood JG, MacIntyre CR. The potential cost-effectiveness of infant pneumococcal vaccines in Australia. *Vaccine* 2011; **29**: 8077–85.
64 Ordóñez JE, Orozco JJ. Cost-effectiveness analysis of the available pneumococcal conjugated vaccines for children under five years in Colombia. *Cost Eff Resour Alloc* 2015; **13**: 6.
65 Bin-Chia Wu D, Chaiyakunapruk N, Chong H-Y, Beutels P. Choosing between 7-, 10- and 13-valent pneumococcal conjugate vaccines in childhood: A review of economic evaluations (2006-2014). *Vaccine* 2015; **33**: 1633–58.
66 Rozenbaum MH, Sanders E a M, van Hoek AJ, *et al.* Cost effectiveness of pneumococcal vaccination among Dutch infants: economic analysis of the seven valent pneumococcal conjugated vaccine and forecast for the 10 valent and 13 valent vaccines. *BMJ* 2010; **340**: c2509.
67 Rubin JL, McGarry LJ, Strutton DR, *et al.* Public health and economic impact of the 13-valent pneumococcal conjugate vaccine (PCV13) in the United States. *Vaccine* 2010; **28**: 7634–43.
68 Sartori AMC, de Soárez PC, Novaes HMD. Cost-effectiveness of introducing the 10-valent pneumococcal conjugate vaccine into the universal immunisation of infants in Brazil. *J Epidemiol Community Health* 2012; **66**: 210–7.
69 Sibak M, Moussa I, El-Tantawy N, *et al.* Cost-effectiveness analysis of the introduction of the pneumococcal conjugate vaccine (PCV-13) in the Egyptian national immunization program, 2013. *Vaccine* 2015; **33**: A182–91.
70 Sohn, S. H, Suh, *et al.* Economic evaluation of childhood 7-valent pneumococcal conjugate

vaccination in Korea. *J Manag Care Pharm* 2010; **16**: 32–45.
71 Strutton DR, Farkouh RA, Earnshaw SR, *et al.* Cost-effectiveness of 13-valent pneumococcal conjugate vaccine: Germany, Greece, and The Netherlands. *J Infect* 2012; **64**: 54–67.
72 Sundaram N, Chen C, Yoong J, *et al.* Cost-effectiveness of 13-valent pneumococcal conjugate vaccination in Mongolia. *Vaccine* 2017; **35**: 1055–63.
73 Talbird SE, Taylor TN, Knoll S, Frostad CR, Martí SG. Outcomes and costs associated with PHiD-CV, a new protein D conjugate pneumococcal vaccine, in four countries. *Vaccine* 2010; **28**: G23–9.
74 Türel Ö, Kisa A, McIntosh EDG, Bakir M. Potential cost-effectiveness of pneumococcal conjugate vaccine (PCV) in Turkey. *Value Heal* 2013; **16**: 755–9.
75 Tyo KR, Rosen MM, Zeng W, *et al.* Cost-effectiveness of conjugate pneumococcal vaccination in Singapore: Comparing estimates for 7-valent, 10-valent, and 13-valent vaccines. *Vaccine* 2011; **29**: 6686–94.
76 Van De Vooren K, Duranti S, Curto A, Garattini L. Cost effectiveness of the new pneumococcal vaccines: A systematic review of European studies. *Pharmacoeconomics* 2014; **32**: 29–45.
77 Urueña A, Pippo T, Betelu MS, *et al.* Cost-effectiveness analysis of the 10- and 13-valent pneumococcal conjugate vaccines in Argentina. *Vaccine* 2011; **29**: 4963–72.
78 van Hoek AJ, Choi YH, Trotter C, Miller E, Jit M. The cost-effectiveness of a 13-valent pneumococcal conjugate vaccination for infants in England. *Vaccine* 2012; **30**: 7205–13.
79 Vučina VV, Filipović SK, Kožnjak N, *et al.* Cost-effectiveness of pneumococcal conjugate vaccination in Croatia. *Vaccine* 2015; **33**: A209–18.
80 Portnoy A, Jit M, Lauer J, *et al.* Estimating costs of care for meningitis infections in low- and middle-income countries. *Vaccine* 2015; **33**: A240–7.
81 Zhang S, Sammon PM, King I, *et al.* Cost of management of severe pneumonia in young children: systematic analysis. *J Glob Health* 2016; **6**: 010408.
82 Aljunid S, Maimaiti N, Ahmed Z, *et al.* Economic Impact of Pneumococcal Protein-D Conjugate Vaccine (PHiD-CV) on the Malaysian National Immunization Programme. *Value Heal Reg Issues* 2014; **3**: 146–55.
83 Blank PR, Szucs TD. Cost-effectiveness of 13-valent pneumococcal conjugate vaccine in Switzerland. *Vaccine* 2012; **30**: 4267–75.
84 Chuck AW, Jacobs P, Tyrell G, Kellner JD. Pharmacoeconomic evaluation of 10- and 13-valent pneumococcal conjugate vaccines. *Vaccine* 2010; **28**: 5485–90.
85 Díez-Domingo J, Ridao-López M, Gutiérrez-Gimeno MV, Puig-Barberá J, Lluch-Rodrigo JA, Pastor-Villalba E. Pharmacoeconomic assessment of implementing a universal PCV-13 vaccination programme in the Valencian public health system (Spain). *Vaccine* 2011; **29**: 9640–8.
86 Duan N. Smearing Estimate : A Nonparametric Retransformation Smearing Estimate : A Nonparametric Retransformation Method. *J Am Stat Assoc* 1983; **78:383**: 605–10.
87 International Labour Organization. Labour force participation rate. Key Indic. Labour Mark. 2015 KILM. 2015. http://www.ilo.org/global/statistics-and-databases/research-and-databases/kilm/WCMS_422090/lang--en/index.htm. (accessed Dec 1, 2016).
88 World Health Organization. National Health Accounts inidicators -Out of Pocket expenditure as % of THE. Glob. Heal. Expend. database. 2014. http://apps.who.int/nha/database/Select/Indicators/en.
89 Tan Torres-Edejer T, Baltussen R, Adam T, Hutubessy R, Acharya A et al. Making choices in health: WHO guide to cost-effectiveness analysis. 2003.
90 Bertram MY, Lauer JA, De Joncheere K, *et al.* Use and misuse of thresholds Cost–

effectiveness thresholds: pros and cons. *WHO Bull* 2016; **94**: 925–30.
91 Newall AT, Jit M, Hutubessy R. Are current cost-effectiveness thresholds for low- and middle-income countries useful? Examples from the world of vaccines. *Pharmacoeconomics* 2014; **32**: 525–31.
92 Woods B, Revill P, Sculpher M, Claxton K. Country-Level Cost-Effectiveness Thresholds: Initial Estimates and the Need for Further Research. *Value Heal* 2016; **19**: 929–35.
